# ICP-MS Elemental Profiling and Antioxidant Characterization of Romanian Artisanal Pălincă-Based Beverages Enriched with Honey, Wild Fruits, and Wood Materials

**DOI:** 10.3390/molecules31152673

**Published:** 2026-07-31

**Authors:** Petrică Tudor Moțiu, Mariana Florica Bei, Ioana Andra Vlad, Szilárd Bartha, Voichița Timiş-Gânsac, Călin Gheorghe Pășcuț, Laviniu Ioan Nuțu Burescu, Eliza Maria Agud, Eugen Traian Jude, Alexandru Ioan Apahidean, Anamaria Călugăr, Adrian Tunduc, Florin Dumitru Bora

**Affiliations:** 1 Department of Forestry and Forest Engineering, University of Oradea, 1 University Street, 410087 Oradea, Romania; tmotiu@uoradea.ro (P.T.M.); sbartha@uoradea.ro (S.B.); vtimis@uoradea.ro (V.T.-G.); cpascut@uoradea.ro (C.G.P.); lburescu@uoradea.ro (L.I.N.B.); 2 Department of Food Engineering, University of Oradea, 1 University Street, 410087 Oradea, Romania; mbei@uoradea.ro (M.F.B.); iavlad@uoradea.ro (I.A.V.); 3 Department of Environmental Engineering, University of Oradea, 1 University Street, 410087 Oradea, Romania; eliza.agud@uoradea.ro (E.M.A.); ejude@uoradea.ro (E.T.J.); 4 Department of Horticulture and Landscape, University of Agricultural Science and Veterinary Medicine of Cluj-Napoca, 3-5 Manastur Street, 400372 Cluj-Napoca, Romania; alexandru.apahidean@usamvcluj.ro; 5 Viticulture and Oenology Department, Advanced Horticultural Research Institute of Transylvania, Faculty of Horticulture and Business in Rural Development, University of Agricultural Science and Veterinary Medicine of Cluj-Napoca, 3–5 Mănăştur Street, 400372 Cluj-Napoca, Romania; anamaria.calugar@usamvcluj.ro; 6 Laboratory of Chromatography, Advanced Horticultural Research Institute of Transylvania, Faculty of Horticulture and Business in Rural Development, University of Agricultural Science and Veterinary Medicine of Cluj-Napoca, 400372 Cluj-Napoca, Romania

**Keywords:** Romanian fruit spirits, multi-element analysis, trace elements, chemometric discrimination, phenolic compounds, radical scavenging capacity, target hazard quotient, hazard index, artisanal beverage processing

## Abstract

Traditional Romanian Pălincă is a protected fruit spirit obtained from fermented fruit matrices, whereas honey- and botanical-enriched Pălincă-based beverages require integrated compositional and technological characterization. This study investigated the physicochemical, chromatic, antioxidant, and ICP-MS elemental profiles of experimental beverages prepared from grape- and plum-based Pălincă distillates enriched with honey, blackthorn, rosehip, or wood-associated materials under controlled artisanal conditions. Chemometric tools were applied to evaluate ingredient- and process-related differentiation, while target hazard quotient and hazard index calculations were retained as a concise screening-level elemental safety assessment. Honey enrichment produced the strongest compositional changes, mainly reflected by increased soluble solids, conductivity, turbidity, chromatic intensity, and mineral content, together with lower final alcoholic strength. Fruit-macerated and mixed honey-botanical beverages were associated with higher phenolic content and antioxidant activity. ICP-MS profiling highlighted potassium as the dominant macroelement, while Cu, Fe, Zn, and Mn were the main technological trace elements. Potentially toxic elements remained at low concentrations, and the screening-level THQ/HI assessment indicated low non-carcinogenic elemental risk under the applied exposure scenario. Overall, the results show that ICP-MS elemental profiling can support the technological and ingredient-related differentiation of experimental Pălincă-based beverages, while toxicological interpretation should remain limited to elemental exposure screening.

## 1. Introduction

“Pălincă” is listed in the European Union eAmbrosia register as a Romanian geographical indication (GI) for a spirit drink and is protected under the EU legal framework governing geographical indications for spirit drinks, including Regulation (EU) 2019/787 [1,2]. According to its product specification, Pălincă is a fruit spirit obtained exclusively through the alcoholic fermentation and distillation of fruit, without the addition of ethyl alcohol of agricultural origin, flavorings, colorants, or sweeteners [2,3,4,5,6]. Romanian artisanal distillates are part of the national gastronomic heritage and are studied for authenticity, composition, multi-element profiles, and elemental safety [7,8,9,10]. Enrichment with honey, wild fruits, and wood materials has generated Pălincă-based beverages with distinct physicochemical, elemental, chromatic, and functional characteristics [11,12,13,14,15].

The elemental composition of alcoholic beverages affects product quality, stability, sensory properties, and consumer safety [16,17,18]. Elevated Fe, Cu, and Zn concentrations may promote oxidation, haze, color changes, and sensory deterioration through interactions with phenolic compounds [19,20,21,22,23,24], while essential elements such as K, Mg, Ca, Cu, Fe, and Zn may support fermentation when present at suitable levels [25,26,27]. Multi-element profiles may reflect raw materials, processing, enrichment, and maturation, but their use for geographical-origin or authenticity assessment requires appropriate sampling and independent validation [28,29,30].

The elemental profile of distilled beverages reflects raw materials, soil and environmental conditions, processing equipment, storage, packaging, and maturation [31,32,33]. Because inorganic elements are generally non-volatile, their direct transfer during distillation is limited; consequently, equipment, dilution water, corrosion, contact materials, storage, packaging, and post-distillation additives may have a greater influence on the final elemental composition [34,35,36,37,38,39,40,41,42]. Honey enrichment, fruit maceration, and wood-assisted maturation may support technological differentiation through chemometric analysis, but geographical-origin and authenticity interpretations require cautious and independently validated assessment [43,44,45].

ICP-MS is a sensitive multi-element technique widely used for beverage analysis, offering low detection limits and a broad linear range [46,47,48,49,50]. Combined with chemometrics, it supports elemental quantification, quality control, and interpretation of compositional differences related to raw materials, processing, maturation, contact materials, and post-distillation treatments [51,52,53,54]. Although applied to wines, beers, spirits, whiskies, vodkas, and fruit distillates, enriched Romanian artisanal Pălincă-based beverages remain insufficiently investigated [55,56,57,58,59,60].

Based on the mineral, phenolic, pigment, and antioxidant composition of honey [61,62,63] and plant-derived materials [64,65], these additives were expected to modify the physicochemical, chromatic, antioxidant, and elemental profiles of Pălincă-based beverages through mineral enrichment, compound extraction, and post-distillation contact effects [66]. This study therefore evaluated these characteristics and the ingredient- and process-related differentiation of experimental beverages produced in Tinca, Bihor County, Romania. A THQ/HI-based screening assessment was also performed to evaluate potential non-carcinogenic elemental concerns under a moderate-consumption scenario.

## 2. Results

### 2.1. Integrated Physicochemical, Chromatic, and Functional Characterization of Experimental Pălincă-Based Beverages

Significant treatment-dependent differences were observed for all physicochemical, chromatic, and functional parameters (*p* < 0.001; Table 1 and Table S1). Honey enrichment decreased alcoholic strength and increased density, electrical conductivity, turbidity, total soluble solids, and dry matter, reflecting the contribution of honey-derived soluble constituents [61]. Fruit maceration mainly affected chromatic and functional parameters, whereas wood-associated variants showed intermediate responses. Mixed honey-botanical variants had the highest total phenolic content and DPPH activity. The strongest treatment effects were observed for total phenolic content, a* coordinate, DPPH inhibition, electrical conductivity, and total soluble solids, as indicated by the F-values and η^2^ values in Table S1.

### 2.2. Major Mineral Composition and Process-Related Elemental Markers

K was the dominant macroelement, followed by Ca, Mg, and Na, while the investigated trace alkali and alkaline-earth elements showed clear processing-dependent variation (Table 2 and Table S2). Control distillates presented the lowest mineral levels, whereas honey-enriched and mixed honey-botanical variants showed the greatest enrichment. All mineral markers differed significantly among variants (*p* < 0.001; Table S2), with the strongest effects observed for Na, K, Rb, Ca, and Mg.

The mineral patterns observed across variants (Table 2 and Table S2) were consistent mainly with post-distillation contributions from honey enrichment, fruit maceration, wood contact, dilution water, equipment, and storage/contact materials, rather than with substantial transfer of non-volatile mineral elements from the original fruit matrix during distillation [17].

### 2.3. Concentrations of Process-, Additive-, and Contact-Related Trace Elements and Potentially Toxic Elements

Fe, Cu, Zn, Mn, and Al showed the clearest processing- and contact-related variability among the investigated trace elements (Table 3 and Table S3). Enriched, macerated, wood-associated, and mixed variants generally differed from the control distillates, but these changes were interpreted as combined effects of additive contribution, post-distillation processing, and contact materials rather than as specific contamination markers. Potentially toxic elements remained at low µg/L concentrations and were therefore considered monitoring parameters rather than indicators of critical contamination.

One-way ANOVA indicated highly significant differences among the experimental variants for all investigated elements (*p* < 0.001; Table S3). 

### 2.4. Screening-Level Elemental Safety Assessment

The THQ/HI assessment was retained as a screening-level elemental safety check. All investigated Pălincă-based beverages showed HI values far below the critical threshold of 1.0 (Table 4 and Table S5), indicating low non-carcinogenic elemental risk under the applied moderate-consumption scenario. Therefore, the small differences observed among variants should be interpreted mainly as treatment-driven elemental variation rather than as toxicological relevance. 

**Figure 1:**
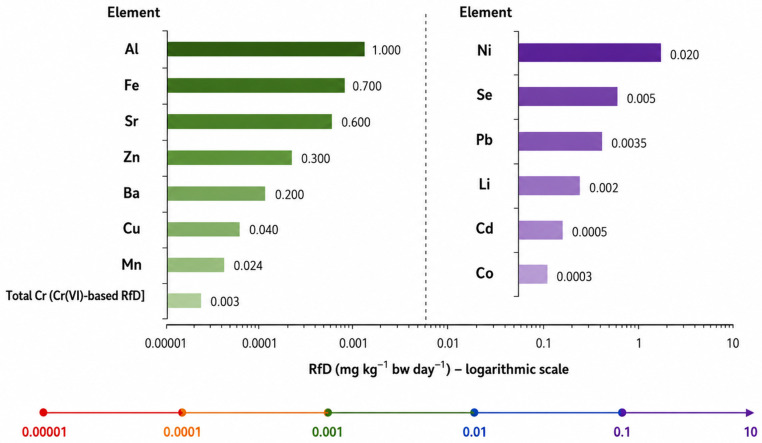
Comparative logarithmic distribution of the oral reference dose (RfD) values applied in the screening-level non-carcinogenic elemental risk assessment of the investigated experimental Pălincă-based beverage variants. The colors and graphical arrangement indicate only the numerical magnitude of the RfD values on a logarithmic scale and do not represent toxicological categories or a universal comparative toxicity ranking.

Because ICP-MS measured total elemental concentrations, the THQ and HI results represent only screening-level risk estimates. Cr was conservatively assessed using the Cr(VI) RfD, whereas As and Hg were excluded from HI because no speciation data were available. The applied RfD values are shown in Figure 1 and Table S5.

### 2.5. Multivariate Chemometric Analysis and PCA-Based Sample Differentiation

**Figure 2:**
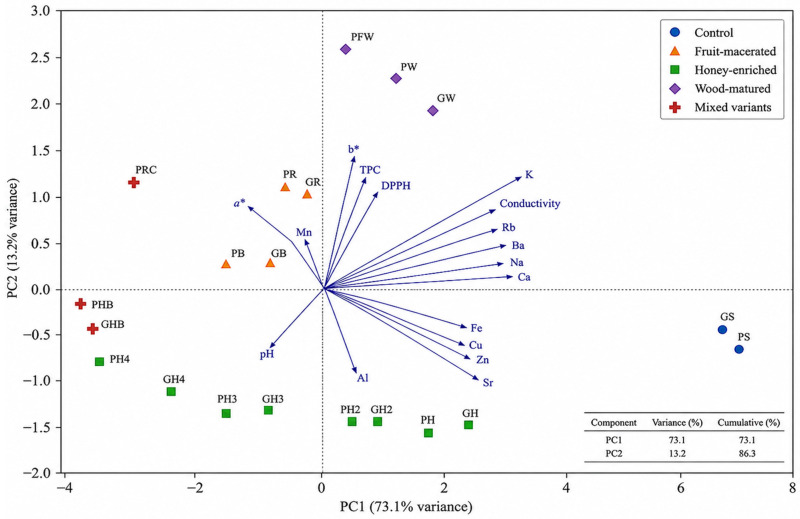
Principal component analysis (PCA) biplot illustrating the chemometric differentiation of experimental Romanian artisanal Pălincă-based beverages based on physicochemical parameters, antioxidant activity, CIELab chromatic coordinates, and ICP-MS elemental composition. The PCA was based on 20 variant-level means, each calculated from three independently prepared replicate beverage samples.

Principal component analysis (PCA) was applied to integrate the physicochemical, chromatic, antioxidant, and ICP-MS elemental data and to evaluate the multivariate differentiation of the experimental Romanian artisanal Pălincă-based beverages. The first two principal components explained 86.3% of the total variance, with PC1 accounting for 73.1% and PC2 for 13.2%, indicating that most of the treatment-related analytical variability was captured by the reduced PCA model (Figure 2).

PCA separated the control distillates from the enriched, macerated, wood-associated, and mixed variants, mainly through chromatic, phenolic, antioxidant, and selected elemental variables (Figure 2). PRC was interpreted as an intensified rosehip-macerated plum variant, PFW as a wood-associated maturation variant, and GHB and PHB as mixed honey–botanical variants. Because the experimental groups were intentionally produced using different treatments, the separation demonstrates process-related compositional differentiation rather than independently validated authentication capacity.

### 2.6. Hierarchical Cluster Analysis (HCA) of Experimental Pălincă-Based Beverage Variants

Hierarchical cluster analysis separated the 20 experimental variant-level means into five treatment-related clusters, summarized in Table 5. Each variant-level mean was calculated from three independently prepared replicate beverage samples. The resulting grouping broadly reflected the main compositional patterns associated with the control distillates, honey enrichment, fruit maceration, intensified rosehip maceration, wood-associated maturation, and combined honey–blackthorn treatment.

Cluster I comprised the traditional grape and plum Pălincă control distillates. Cluster II included all honey-enriched grape- and plum-based variants. Cluster III comprised the simple fruit-macerated variants together with PRC, which showed a fruit-associated compositional profile because it was prepared through intensified maceration with fragmented rosehips. Cluster IV included the three wood-associated maturation variants, including PFW, which was prepared exclusively with mulberry wood fragments and without added fruit material. Cluster V comprised GHB and PHB, representing the mixed honey–blackthorn treatments.

The HCA grouping was broadly consistent with the PCA results and reflected the principal physicochemical, chromatic, functional, and elemental effects of the applied treatments. However, the clusters represent empirical statistical groupings within the investigated dataset and are not fully identical to the a priori technological categories. Accordingly, the observed clustering supports process-related compositional differentiation but does not constitute independent evidence of authentication capacity.

Hierarchical cluster analysis (HCA) was performed on the standardized variant-level mean values using Euclidean distance and Ward’s linkage method. A five-cluster exploratory solution was retained based on the interpretability of the treatment-related grouping, and the resulting cluster membership is summarized in Table 5.

### 2.7. Correlation Network Analysis of Physicochemical, Functional, and Elemental Parameters

**Figure 3:**
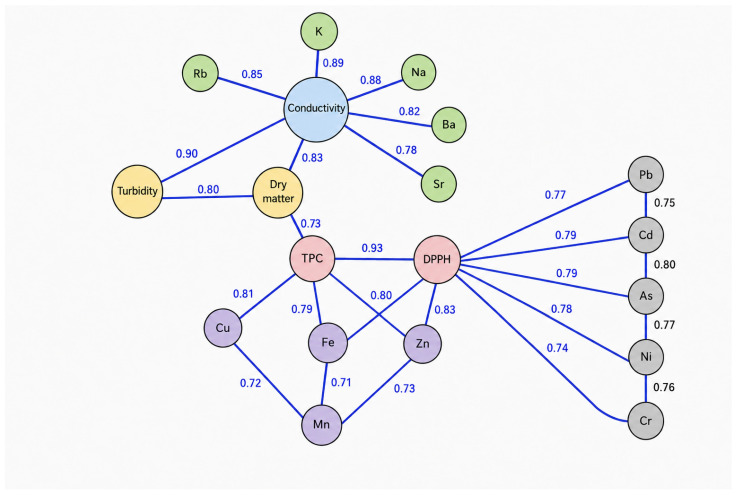
Pearson correlation network of functional, physicochemical, and elemental parameters in experimental Pălincă-based beverage variants. The network was constructed using 20 variant-level means, each obtained by averaging the three independently prepared replicate beverage samples corresponding to each experimental variant. Only strong Pearson correlations with |r| > 0.70 and Benjamini–Hochberg false-discovery-rate-adjusted *p* < 0.05 were retained. Both positive and negative correlations were considered, and their direction is distinguished by the edge coding shown in the figure. Edge thickness is proportional to the absolute magnitude of the correlation coefficient. Correlations represent statistical co-variation within the experimental dataset and should not be interpreted as evidence of causal or mechanistic relationships. Node colors indicate the parameter categories: electrical conductivity (light blue), other physicochemical parameters (yellow), functional parameters (pink), major and trace elements associated with mineral composition (green), process- and contact-related trace elements (purple), and potentially toxic elements (gray).

The correlation network identified two principal association domains (Figure 3). The mineral–conductivity domain linked conductivity with K, Na, Rb, Ba, Sr, dry matter, and turbidity, whereas the functional domain was characterized by the strong TPC–DPPH association (r = 0.93). Correlations between antioxidant parameters and Cu, Fe, Zn, or Mn were interpreted as treatment-driven co-variation rather than direct antioxidant effects. Similarly, associations among potentially toxic elements indicated shared variation within the experimental dataset and not necessarily direct physicochemical interactions.

After adjustment for multiple testing using the Benjamini–Hochberg false discovery rate procedure, the correlation network retained associations with |r| > 0.70 and adjusted *p* < 0.05. Therefore, both strong positive and strong negative correlations were eligible for inclusion in the network. The main association domains remained represented by the relationships between electrical conductivity and mineral-related variables and by the strong positive association between TPC and DPPH activity.

## 3. Discussion

### 3.1. Effect of Honey Enrichment on Physicochemical, Functional, and Elemental Characteristics

**Figure 4:**
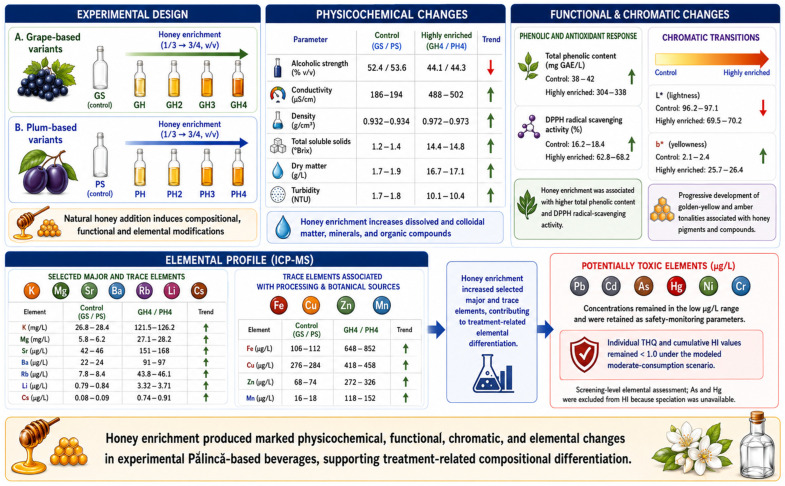
Integrated effects of honey enrichment on the physicochemical, functional, and screening-level elemental exposure profile of Pălincă-based beverages. Red downward arrows indicate decreases, whereas green upward arrows indicate increases in the highly honey-enriched variants compared with the corresponding controls.

Honey enrichment produced the strongest compositional changes, primarily through dilution of the distillate and incorporation of sugars, colloidal constituents, pigments, phenolic compounds, and minerals. These contributions explain the lower alcoholic strength and the concurrent increases in soluble solids, density, conductivity, turbidity, phenolic content, antioxidant activity, and yellow–amber coloration (Table S6 and Figure 4), in agreement with previous reports on honey matrices [61,62].

The associated elemental enrichment, characterized mainly by increased K, Mg, Sr, Ba, Rb, Li, and Cs, together with moderate increases in Fe, Cu, Zn, and Mn, was consistent with the contribution of honey-derived inorganic constituents [63] (Table S14). However, because the honey was not analyzed separately, direct source-specific attribution remains tentative. Its elemental composition may also reflect soil and geochemical background, atmospheric deposition, water availability, and other environmental factors; therefore, the observed changes should be interpreted as additive-associated enrichment rather than as a botanical-origin marker.

### 3.2. Effect of Fruit Maceration on Chromatic, Antioxidant, and Elemental Differentiation

Fruit maceration modified the chromatic and functional profiles through extraction of pigments, phenolic compounds, soluble fractions, and colloidal material. Blackthorn variants showed predominantly reddish coloration consistent with anthocyanin- and tannin-associated contributions, whereas rosehip variants showed stronger yellow–orange development associated with carotenoid-like pigments and oxidized phenolics [64,65]. The stronger response of PRC was consistent with enhanced extraction resulting from increased contact surface.

The elemental changes observed after maceration were compatible with botanical and contact-associated contributions. However, they may also reflect soil properties, geochemical background, plant uptake, environmental exposure, and extraction efficiency. Consequently, these profiles should be interpreted as maceration-associated compositional patterns rather than definitive evidence of element transfer from the fruit material alone.

### 3.3. Effect of Wood-Associated Processing on Elemental Composition and Product Differentiation

Wood-associated processing produced a more moderate compositional response than honey enrichment or fruit maceration. The development of yellow–brown and amber tones, together with moderate increases in TPC and DPPH activity, was consistent with the gradual extraction of wood-associated phenolic compounds, tannin-like constituents, lignin degradation products, and oxidation-related compounds [66] (Table S8).

Elemental variation was also consistent with wood- and contact-associated contributions [33] (Table S8), although direct wood-derived transfer cannot be confirmed because the wood materials were not separately analyzed. PFW was prepared by maturation of plum Pălincă with mulberry wood fragments and should therefore be interpreted as a wood-associated maturation variant. However, because the mulberry wood material and the corresponding pre-contact beverage matrix were not analyzed separately, the observed compositional changes cannot be attributed exclusively to the wood material. Accordingly, the observed profile supports treatment-related differentiation but not direct inference of superior quality or source-specific composition.

### 3.4. ICP-MS Mineral Profiling and Technological Differentiation

ICP-MS profiling showed that the control distillates represented a low-mineral baseline, consistent with the limited transfer of non-volatile inorganic elements during distillation, whereas the enriched, macerated, wood-associated, and mixed variants showed treatment-associated inorganic shifts [17,67] (Table S9). Among the major elements, K, Ca, Mg, and Na mainly described additive- and contact-associated ionic load, reflecting the contribution of natural ingredients and maturation materials to the inorganic composition of the beverages. In parallel, Rb, Sr, and Ba provided more informative ingredient- and raw-material-related signals because they are more closely linked to plant-derived, soil–water, and raw-material inputs than to metallic equipment contact [17,59,67].

In the present dataset, K, Rb, Sr, and Ba showed clearer ingredient- and process-related differentiation than Fe and Cu. Potassium and Rb may reflect botanical and geochemical contributions, whereas Sr and Ba are commonly associated with soil–water–raw material interactions. By contrast, Fe, Cu, Zn, and Mn are more strongly affected by traditional processing conditions, distillation equipment, metallic fittings, contact materials, storage conditions, and oxidative transformations. Therefore, these elements should be interpreted primarily as technological and process-control indicators rather than as source-specific traceability markers [59,67].

Overall, the observed elemental profiles should be considered treatment-associated differentiation patterns. Because the additives, wood materials, dilution water, and pre-contact matrices were not analyzed separately, the exact origin of individual elements cannot be established. These profiles therefore support ingredient- and process-related interpretation within the investigated experimental set, but not definitive geographical-origin, authenticity, or traceability claims.

### 3.5. Interpretation of Process-, Additive-, and Contact-Related Trace Elements and Potentially Toxic Elements

Trace-element concentrations varied among the investigated Pălincă-based beverages, reflecting the combined influence of the original distillate matrix, post-distillation processing, botanical additives, wood contact, storage materials, environmental background, and possible interaction with technological equipment [68,69,70] (Tables S10 and S11). Fe, Cu, Zn, and Mn were therefore interpreted primarily as process- and contact-related indicators rather than as markers of authenticity.

Cu and Fe may originate from copper stills, metallic fittings, contact surfaces, storage materials, additives, or oxidation-related processes, whereas variation in Zn and Mn may additionally reflect contributions from botanical materials and other ingredients [68,69,70]. Because these potential sources were not analyzed separately, the observed increases in enriched, macerated, wood-associated, and mixed variants cannot be attributed to a single technological or additive source.

Pb, Cd, As, Hg, Ni, and Cr were retained as safety-monitoring parameters. Although their concentrations showed treatment-related variation, the measured values were within or below ranges commonly reported for distilled beverages and did not indicate excessive elemental contamination [29,68,69,70]. Nevertheless, concentration comparisons alone are insufficient for toxicological interpretation; therefore, their safety relevance was evaluated using the exposure-based THQ and HI assessment.

### 3.6. Non-Carcinogenic Risk Characterization Based on THQ and HI Assessment

The THQ/HI assessment integrated the quantified elemental concentrations, toxicological reference values, and exposure assumptions to provide an exposure-based risk characterization under the modeled moderate-consumption scenario [69,71,72]. Elements with lower reference dose values may contribute more strongly to cumulative risk even when present at relatively low concentrations. All individual THQ values and cumulative HI values remained below 1.0, indicating a low non-carcinogenic risk for the elements included in the model (Table S12). The slightly higher values observed in enriched and mixed variants reflected cumulative elemental inputs associated with the additives and processing conditions but remained below the toxicological threshold of concern. This interpretation is restricted to the quantified elemental fraction and should not be extrapolated to ethanol, methanol, ethyl carbamate, volatile contaminants, or the overall safety of the beverages.

### 3.7. Chemometric Interpretation and Correlation-Based Differentiation

PCA, HCA, and correlation analysis integrated the physicochemical, chromatic, functional, and elemental variables into coherent treatment-related patterns [67] (Figure 2 and Figure 3; Table 5 and Table S13). The separation primarily reflected the contrast between the low-mineral controls, honey-associated soluble-solid and mineral enrichment, fruit-associated chromatic and phenolic changes, and more moderate wood/contact-related effects.

Because the sample groups were intentionally produced using different processing procedures, their separation was expected and should not be interpreted as evidence of validated ICP-MS authentication capacity. The mixed and intensified variants also require cautious interpretation: An integrated summary of the technological approaches, representative variants, associated constituents, principal observed outcomes, and the clarified classification of PRC and PFW is provided in Table 6. PRC represents intensified rosehip maceration, whereas GHB and PHB reflect cumulative honey–botanical effects. PFW represents a wood-associated maturation variant prepared from plum Pălincă matured with mulberry wood fragments. Their chemometric separation therefore supports integrated process-related differentiation, but not strict source-specific attribution or validated authentication of these confounded formulations (Table S14).

The findings should also be interpreted within the limits of this preliminary, product-specific study, which included beverages produced in a single region and season without external validation, commercial or adulterated reference samples, separately characterized additives and initial distillates, isotopic confirmation, or predictive authentication models. The observed profiles therefore represent ingredient- and process-related patterns within the investigated experimental set rather than definitive markers of geographical origin, formal authenticity, or complete traceability. Future validation should include independent multi-season batches, commercially representative samples, characterized raw materials, and complementary isotopic, volatile, sensory, microbiological, and safety analyses. In addition, although false discovery rate adjustment was applied to the correlation analysis, no global multiplicity correction was applied across the separate univariate ANOVA models conducted for the different analytical endpoints. Therefore, some inflation of the overall type I error rate across endpoints cannot be completely excluded, and the univariate findings should be interpreted together with their effect sizes, treatment-related consistency, and multivariate patterns.

## 4. Materials and Methods

### 4.1. Study Area and Experimental Design

The study was conducted during the 2025 production season on traditional Pălincă distillates and experimental Pălincă-based beverages produced in Bihor County, north-western Romania. The experimental design is summarized in Table 7, while additional processing, quality-control, traceability, and analytical-allocation details are provided in Figure S1.

All raw materials used in this experimental series were obtained from the same geographical area in order to maintain a common regional production context. This choice was not intended to infer legal authenticity or terroir-based origin attribution for the enriched Pălincă-based beverages. Grapes and plums used for distillate production originated from the Tinca area and adjacent locations within Bihor County, Romania, while blackthorn fruits (*Prunus spinosa* L.) and rosehips (*Rosa canina* L.) were collected from local orchard margins and spontaneous vegetation. Prior to processing, all plant materials were visually inspected, and damaged, contaminated, or insufficiently matured materials were excluded to ensure comparable raw-material quality across the experimental variants.

The experimental series included traditional grape- and plum-based control distillates, honey-enriched variants, fruit-macerated variants, wood-associated variants, and mixed or intensified botanical variants, all prepared under comparable artisanal conditions. This design allowed the evaluation of compositional changes associated with honey addition, fruit maceration, wood contact, and combined botanical processing while maintaining a common regional production context.

For terminology consistency, the term “Pălincă” is used in the present study only for the traditional grape- and plum-based fruit distillates used as control samples or base matrices. All products obtained after honey enrichment, fruit maceration, wood-associated processing, or combined botanical treatment are hereafter referred to as experimental Pălincă-based beverages or as beverages prepared from traditional Pălincă distillates, rather than as Pălincă, to distinguish them from the legally defined spirit drink registered under the European Union geographical indication (GI) scheme. Residual distillation fractions were excluded from the analytical dataset.

### 4.2. Experimental Replication Strategy and Sample Size Justification

The experimental design included 20 Pălincă-based beverage variants, each represented by three independently processed replicate beverage samples (*n* = 3), resulting in 60 experimental samples. For each variant, the three replicate beverage samples were obtained from separate distillate aliquots and processed independently under identical variant-specific conditions, including the same additive proportion and maceration/maturation period. These independently processed replicate beverage samples were considered the experimental units for statistical analysis. For each sample, triplicate instrumental readings were performed and averaged before statistical analysis; these instrumental readings were used only to assess analytical repeatability and were not treated as independent experimental replicates.

Control samples consisted of traditional grape and plum Pălincă distillates (GS and PS). The remaining variants were prepared from grape- or plum-based Pălincă distillates enriched with honey, wild fruits, wood materials, or mixed honey-botanical additives under standardized artisanal conditions. Detailed experimental classification and coding of all investigated Pălincă-based beverages are presented in Supplementary Table S15.

### 4.3. Raw Materials Used for the Production of Traditional Pălincă Distillates and Pălincă-Based Beverages

The raw materials used in the present study included grapes (*Vitis vinifera* L.), plums (*Prunus domestica* L.), blackthorn fruits (*Prunus spinosa* L.), rosehips (*Rosa canina* L.), natural honey, and plum and mulberry wood fragments used for maturation procedures. Damaged or microbiologically altered materials were excluded prior to processing.

### 4.4. Production Technology of the Traditional Pălincă Distillates Used as Base Matrices

Fresh grapes (*Vitis vinifera* L.) and plums (*Prunus domestica* L.) originating from the Tinca area (Bihor County, north-western Romania) were processed under standardized artisanal production conditions prior to fermentation and distillation. Fermentation and double distillation were performed using comparable physicochemical, chromatic, and functional parameters for all experimental variants in order to ensure experimental consistency and comparability between samples. Following distillation, the obtained grape- and plum-based distillates were used for honey enrichment, fruit maceration, wood-assisted maturation, and mixed flavoring procedures under standardized artisanal processing conditions. Detailed technological conditions and experimental preparation parameters are summarized in Supplementary Tables S15–S17.

### 4.5. Collection, Pre-Treatment, and Standardization of Additives Used for Experimental Pălincă-Based Beverages

Natural additives used in the present study included artisanal honey, blackthorn fruits (*Prunus spinosa* L.), rosehips (*Rosa canina* L.), and plum or mulberry wood fragments used for maturation procedures. All additives originated from the Tinca area (Bihor County, north-western Romania) and were prepared under comparable artisanal and laboratory conditions prior to incorporation into the distillates. The honey used for enrichment was a locally produced artisanal polyfloral honey from the Tinca area. According to the producer declaration, the honey was obtained from mixed local floral sources; however, its botanical origin was not confirmed by melissopalynological analysis. Therefore, honey-related interpretations were restricted to additive-associated compositional effects rather than definitive botanical-origin attribution. Detailed additive preparation procedures, enrichment ratios, fragmentation characteristics, and maceration parameters are summarized in Supplementary Tables S15–S17.

### 4.6. Preparation of Honey-Enriched, Fruit-Macerated, Wood-Associated, and Mixed Pălincă-Based Beverages

The experimental Pălincă-based beverages were prepared from traditional grape and plum Pălincă distillates obtained by double distillation under artisanal conditions. The untreated control distillates GS and PS were maintained without additive incorporation and analyzed at their original alcoholic strengths. For the preparation of the honey-enriched, fruit-macerated, wood-associated, and mixed/intensified botanical variants, separate grape- and plum-based distillate aliquots were adjusted to a target alcoholic strength of approximately 50% *v*/*v* before additive incorporation. The treated aliquots were subsequently enriched with honey, wild fruits, wood fragments, or combined honey-botanical/intensified botanical materials. Detailed processing conditions, additive proportions, maturation parameters, and sample classification are provided in Supplementary Tables S15 and S16.

For statistical and comparative interpretation, the experimental variants were assigned a priori to predefined technological categories: control distillates (GS and PS), honey-enriched variants (GH–GH4 and PH–PH4), simple fruit-macerated variants (GB, GR, PB, and PR), wood-associated variants (GW, PW, and PFW), and mixed/intensified botanical variants (PRC, GHB, and PHB). PRC was treated as an intensified rosehip-macerated plum variant and was therefore grouped within the mixed/intensified botanical category, although its fruit-like compositional features were discussed comparatively with the simple fruit-macerated variants. PFW was prepared from plum Pălincă matured with mulberry wood fragments, without the addition of fruit material, and was classified as a wood-associated maturation variant. GHB and PHB were classified as mixed honey-botanical variants and were not pooled with the simple honey-enriched or simple fruit-macerated groups for category-level interpretation.

For reproducibility, the exact variant-specific additive proportions and maceration/maturation periods applied to each experimental code are reported in Supplementary Table S16. These conditions were fixed within each variant and were applied consistently to three separately prepared distillate aliquots. Accordingly, each of the 20 variants was represented by three independently prepared replicate beverage samples (*n* = 3), resulting in 60 independent experimental samples. Triplicate instrumental readings were averaged for each sample before statistical analysis and were not treated as independent experimental replicates.

### 4.7. Physicochemical, Antioxidant, and Chromatic Analyses

#### 4.7.1. Alcoholic Strength (% *v*/*v*)

Alcoholic strength was determined densimetrically from the distilled fraction of each beverage sample, in order to avoid interference from sugars, soluble extractives, suspended material, and other non-volatile compounds present in honey-enriched, fruit-macerated, wood-associated, and mixed variants. Briefly, each sample was distilled, and the obtained distillate was brought to 20 °C before measurement. Alcoholic strength was then determined using a DA-130N Portable Density/Specific Gravity Meter (Kyoto Electronics Manufacturing Co., Ltd., Kyoto, Japan), and results were expressed as percentage alcohol by volume (% *v*/*v*). The density values reported separately for the final beverages were measured directly on the original samples and were not used as a substitute for alcoholic-strength determination.

#### 4.7.2. pH Determination

pH values were measured using a SevenCompact S220 pH meter (Mettler-Toledo AG, Greifensee, Switzerland) calibrated with standard buffer solutions at pH 4.00 and 7.00.

#### 4.7.3. Electrical Conductivity (µS/cm)

Electrical conductivity was determined using a SevenCompact S230 conductivity meter (Mettler-Toledo AG, Greifensee, Switzerland) equipped with automatic temperature compensation. Results were expressed as µS/cm.

#### 4.7.4. Density (g/cm^3^)

Density was determined using a DMA 35 Basic digital densimeter (Anton Paar GmbH, Graz, Austria) operating on the oscillating U-tube principle. Results were expressed as g/cm^3^.

#### 4.7.5. Turbidity (NTU)

Turbidity was evaluated nephelometrically using an HI88703 turbidimeter (Hanna Instruments Inc., Woonsocket, RI, USA). Results were expressed as nephelometric turbidity units (NTU).

#### 4.7.6. Total Soluble Solids (°Brix)

Total soluble solids were determined using a PAL-1 digital refractometer (ATAGO Co., Ltd., Tokyo, Japan). Results were expressed as °Brix.

#### 4.7.7. Dry Matter Content (g/L)

Dry matter content was determined gravimetrically after thermal drying at 105 °C until constant mass. Results were expressed as g/L.

#### 4.7.8. Total Phenolic Content (TPC)

Total phenolic content was determined spectrophotometrically using the Folin–Ciocalteu method at 765 nm with gallic acid as calibration standard. Results were expressed as mg gallic acid equivalents per liter (mg GAE/L).

#### 4.7.9. DPPH Radical Scavenging Activity

Antioxidant activity was evaluated using the DPPH radical scavenging assay at 517 nm using a UV–Vis spectrophotometer (UV-1900i, Shimadzu Corporation, Kyoto, Japan). Antioxidant activity was expressed as percentage inhibition according to the following equation:

$$DPPH\;inhibition\;(\%)=\frac{A_{control}-A_{sample}}{A_{control}}\times 100$$

### 4.8. ICP-MS Multi-Elemental Analysis

Multi-elemental analysis was performed using an iCAP Q ICP-MS system (Thermo Fisher Scientific, Bremen, Germany) operated in He-KED mode, after microwave-assisted acid digestion with ultrapure nitric acid and hydrogen peroxide. Microwave-assisted sample digestion was carried out using a Milestone closed-vessel microwave digestion system (Milestone Srl, Sorisole, Italy) equipped with hermetically sealed PTFE digestion vessels. External calibration and internal standard correction were used for the quantitative determination of K, Ca, Mg, Na, Al, Fe, Cu, Zn, Mn, Co, Se, Sr, Ba, Rb, Li, Cs, Pb, Cd, As, Hg, Ni, and Cr. Each independent sample was analyzed in triplicate instrumental readings, which were averaged before statistical analysis. A complete ICP-MS validation dataset, including calibration range, linearity, internal standards, LoD, LoQ, BEC, recovery, intra-day and inter-day precision, expanded uncertainty, interference-control strategy, instrumental operating conditions, QA/QC sequence, and CRM verification, is provided in Supplementary Tables S18–S22.

### 4.9. Quality Assurance and Quality Control (QA/QC)

Quality assurance and quality control procedures were applied throughout the ICP-MS analysis to ensure analytical accuracy, repeatability, and instrumental stability. Procedural blanks, calibration verification standards, certified reference materials, recovery assessment, precision control, and instrumental drift monitoring were included in the analytical sequence. Method performance was evaluated based on linearity, limits of detection and quantification, recovery values, relative standard deviation, and CRM agreement. Detailed analytical validation parameters and QA/QC criteria are summarized in Supplementary Tables S19–S22.

### 4.10. Chromatic Characterization and Qualitative Visual Assessment

Chromatic characterization of the experimental artisanal Pălincă-based beverages was performed using the CIELab color system with a portable colorimeter (CR-400 Chroma Meter, Konica Minolta, Osaka, Japan). The evaluated parameters included L*, a*, and b*. For each independently processed replicate beverage sample, triplicate instrumental readings were recorded and averaged before statistical processing. Results were expressed as the mean ± standard deviation of three independently processed replicate beverage samples per experimental variant (*n* = 3). Qualitative visual observations were additionally performed under standardized laboratory conditions to support chromatic characterization. No formal sensory panel or quantitative sensory evaluation was included in the present study.

### 4.11. Toxicological Exposure Assessment and Human Health Risk Characterization

A screening-level non-carcinogenic elemental safety assessment was performed using Estimated Daily Intake (EDI), Target Hazard Quotient (THQ), and Hazard Index (HI), following commonly applied dietary exposure assessment approaches.

The Estimated Daily Intake (EDI) was calculated as:

$$EDI=\frac{C\times DI}{BW}$$

where EDI = estimated daily intake on exposure days (mg/kg body weight/day); C = concentration of the investigated element in the analyzed beverage (mg/L); DI = intake rate of the alcoholic beverage per exposure day (L/exposure day); BW = body weight (kg).

The Chronic Daily Intake (CDI) was calculated as:

$$CDI=\frac{C\times DI\times EF\times ED}{BW\times AT}$$

where CDI = chronic daily intake (mg/kg body weight/day); C, DI, and BW have the same meanings as defined for EDI; EF = exposure frequency (days/year); ED = exposure duration (years); AT = averaging time (days).

For adult exposure assessment, DI was set at 0.05 L/day, corresponding to an assumed moderate consumption of 50 mL/day, with EF = 365 days/year, ED = 30 years, BW = 70 kg, and AT = EF × ED. Under these assumptions, CDI was mathematically equivalent to EDI and was therefore not interpreted as a separate exposure metric; THQ and HI were used for non-carcinogenic risk characterization.

The Target Hazard Quotient (THQ) was calculated using:

$$THQ=\frac{CDI}{RfD}$$

where THQ = target hazard quotient; RfD = oral reference dose (mg/kg body weight/day).

The cumulative non-carcinogenic risk was expressed as the Hazard Index (HI), calculated as the sum of the individual Target Hazard Quotient (THQ) values for the elements included in the risk model:

*HI* = ∑*THQ_i_*

where HI represents the cumulative hazard index and THQᵢ represents the target hazard quotient of each investigated element. 

RfD values used for THQ and HI calculations were selected from EPA, WHO/FAO, ATSDR, and related toxicological guidance sources, as indicated in Table S5. Since ICP-MS quantified total elemental concentrations, the THQ/HI assessment was interpreted as a conservative screening-level non-carcinogenic elemental risk estimate and did not account for chemical speciation. Total Cr was assessed conservatively using a Cr(VI)-based RfD, whereas total As and total Hg were reported analytically but excluded from the cumulative HI calculation due to the absence of speciation data. THQ and HI values below 1 were considered indicative of low non-carcinogenic elemental risk under the applied exposure scenario.

### 4.12. Statistical Analysis

Results were expressed as the mean ± standard deviation of three independently prepared replicate beverage samples per experimental variant (*n* = 3). The three instrumental readings obtained for each independently prepared sample were averaged before statistical analysis and were not considered separate experimental units. Differences among the 20 experimental variants were evaluated using one-way analysis of variance (ANOVA), followed by Duncan’s multiple range test, with statistical significance set at *p* < 0.05. Data normality and homogeneity of variance were assessed using the Shapiro–Wilk and Levene tests, respectively.

For each analyzed parameter, the effect size associated with the experimental variant was expressed as eta squared (η^2^) and calculated from the one-way ANOVA as:

*H*^2^ = *SS_between*/*SS_total*

where SS_between represents the sum of squares attributable to differences among the experimental variants and SS_total represents the total sum of squares calculated from the 60 independent experimental samples. Eta squared was used to quantify the proportion of the total variability explained by the experimental variant. 

The overall coefficient of variation for each parameter was calculated as:

$$\mathrm{CV}(\%)=(\mathrm{SD}\text{\_}\mathrm{overall}/\bar{x}\text{\_}\mathrm{overall})\times 100$$

where SD_overall and $\bar{x}\text{\_}\mathrm{overall}$ represent the overall standard deviation and grand arithmetic mean, respectively, calculated from the 60 independent experimental samples. Instrumental replicate readings were averaged before calculation and were not treated as independent observations. The coefficient of variation was used to express the overall variability of each parameter relative to its grand mean. The CV was not calculated for the a* coordinate because the dataset included both negative and positive values, making a relative dispersion measure based on the arithmetic mean inappropriate.

Pearson correlation analysis, principal component analysis (PCA), hierarchical cluster analysis (HCA), and correlation network analysis were performed on the integrated physicochemical, chromatic, functional, and ICP-MS elemental dataset. For these multivariate analyses, the three independently prepared replicate samples corresponding to each experimental variant were averaged. Therefore, the observational unit was the variant-level mean, and the analyses were performed using 20 variant-level means (*n* = 20), rather than the 60 individual experimental samples. Variant-level mean values were standardized by z-score transformation before multivariate analysis. Hierarchical cluster analysis (HCA) was performed on the standardized variant-level mean values using Euclidean distance and Ward’s linkage method. No conversion of linkage distances into similarity percentages was performed. Rather than applying a similarity-percentage cut-off, the Ward hierarchy was partitioned into five clusters, and this exploratory solution was retained based on the interpretability and internal coherence of the treatment-related grouping.

To account for multiple testing in the correlation matrix, the raw p-values associated with the Pearson correlation coefficients were adjusted using the Benjamini–Hochberg false discovery rate procedure. The correlation network retained only strong associations with |r| > 0.70 and false-discovery-rate-adjusted *p*-values < 0.05. The use of the absolute correlation coefficient allowed both strong positive and strong negative associations to be considered. The correlation network was interpreted as an exploratory visualization and not as evidence of causal or mechanistic relationships.

No additional multiplicity correction was applied across the separate one-way ANOVA models conducted for the different analytical parameters. Duncan’s multiple range test was used for post hoc comparisons among experimental variants within each analyzed parameter. The absence of a global multiplicity correction across all univariate endpoints represents a limitation of the study and may increase the risk of Type I error; therefore, the corresponding statistical findings were interpreted cautiously.

Statistical analyses were performed using XLSTAT (Lumivero, Denver, CO, USA), IBM SPSS Statistics (IBM Corp., Armonk, NY, USA), and Microsoft Excel 365 (Microsoft Corporation, Redmond, WA, USA).

## 5. Conclusions

This study showed that the investigated Romanian artisanal Pălincă-based beverages were differentiated according to the applied enrichment, maceration, wood-contact, and mixed processing procedures. Honey enrichment was associated with the strongest compositional changes, including increased °Brix, conductivity, dry matter, turbidity, TPC, DPPH activity, and mineral content, especially K, Ca, Mg, Na, Sr, Ba, Rb, Fe, Cu, Zn, and Mn. Fruit maceration was associated mainly with chromatic intensification, changes compatible with phenolic extraction, antioxidant response, and trace-element differentiation, while wood-associated processing showed a more moderate but distinct elemental and chromatic profile. The mixed variants showed the highest compositional complexity, suggesting cumulative effects of combined enrichment and maceration procedures.

ICP-MS profiling supported the differentiation of process-related compositional patterns among control, honey-enriched, fruit-macerated, wood-associated, and mixed Pălincă-based beverages, particularly through the variation of K, Ca, Mg, Na, Sr, Ba, Rb, Li, Cs, Fe, Cu, Zn, and Mn. However, because the experimental groups were intentionally prepared using markedly different enrichment, maceration, wood-contact, and mixed processing procedures, the observed multivariate separation should be regarded as evidence of controlled process-related compositional differentiation rather than as definitive proof of ICP-MS-based authentication capacity. Further studies including independent, naturally variable, and commercially representative sample sets are necessary to validate these elemental fingerprints as authentication or traceability markers.

However, the observed elemental patterns should be interpreted as process- and ingredient-related profiles modulated by soil/geochemical background and environmental factors, especially for honey and fruit-derived additives, rather than as markers determined exclusively by botanical origin.

The THQ/HI assessment was retained only as a screening-level elemental safety check for the metals and trace elements quantified by ICP-MS in the investigated beverage matrices. All individual THQ and cumulative HI values remained below 1.0, indicating low non-carcinogenic elemental risk under the modeled moderate-consumption scenario. However, these results should not be interpreted as a complete toxicological, food-safety, or quality evaluation of the beverages, because ethanol-related exposure was not included, and methanol, ethyl carbamate, volatile contaminants, pesticide residues, microbiological parameters, isotope-ratio authentication data, and formal sensory descriptors were not assessed. Therefore, the safety interpretation of the present study is restricted exclusively to the quantified elemental fraction under the applied exposure assumptions.

## Figures and Tables

**Table 1:** Summary Statistics, Effect Sizes, and Treatment-Related Trends in the Physicochemical, Chromatic, and Functional Parameters of Experimental Pălincă-Based Beverages.

Parameter	Approximate Observed Range	Overall Mean	η^2^	CV (%)	Main Treatment-Related Pattern
Alcoholic strength (% *v*/*v*)	≈36.0–53.6	47.92	0.78	5.71	Highest in controls; generally decreased after enrichment or maceration
pH	3.72–4.34	4.00	0.69	4.06	Relatively limited variation; slight decrease in several enriched and mixed variants
Electrical conductivity (µS/cm)	186–502	388.5	0.86	23.84	Lowest in controls; markedly increased after additive incorporation and maturation
Density (g/cm^3^)	0.932–0.973	0.956	0.81	1.32	Increased in most treated variants, particularly after enrichment and maceration
Turbidity (NTU)	1.7–10.6	7.11	0.84	39.72	Lowest in controls; increased substantially in enriched, fruit-associated, wood-associated, and mixed variants
Total soluble solids (°Brix)	1.2–14.8	8.67	0.87	51.38	Strongly increased following honey enrichment and botanical maceration
L*	46.8–97.1	69.01	0.82	20.41	Highest in controls; generally decreased as chromatic intensity increased
a*	−2.1–29.1	8.72	0.89	n.a.	Strongest positive values in fruit-associated and intensified botanical variants
b*	2.1–38.2	20.41	0.85	48.96	Increased mainly in rosehip-associated, intensified, and selected wood-associated variants
Dry matter (g/L)	1.7–17.1	11.12	0.84	40.88	Lowest in controls; increased after honey addition and botanical processing
Total phenolic content (mg GAE/L)	38–648	338.4	0.89	44.15	Highest responses occurred in fruit-associated, wood-associated, and mixed/intensified variants
DPPH inhibition (%)	16.2–82.4	59.4	0.87	27.63	Lowest in controls; increased substantially after fruit, wood, honey, and combined treatments

Note: Values represent the approximate minimum–maximum interval and overall mean displayed in the original multi-panel figure. All treatment effects were statistically significant at *p* < 0.001. The coefficient of variation was not considered informative for a* because the dataset included both negative and positive values around zero. Values were obtained from three independently prepared beverage samples per experimental variant (*n* = 3), with instrumental replicate readings averaged before statistical analysis. n.a., not applicable. The coefficient of variation (CV) was not calculated for a* because the dataset included both negative and positive values; therefore, CV would not provide a meaningful measure of relative variability.

**Table 2:** Concentrations of major and trace alkali and alkaline earth elements in the analyzed spirit beverage groups and the number of independent samples included in each technological category.

Sample Codes	Analyzed Samples (*n*)	K mg/L	Ca mg/L	Mg mg/L	Na mg/L	Sr µg/L	Ba µg/L	Rb µg/L	Li µg/L	Cs µg/L
GS, PS	6	27.6 (26.8–28.4)	12.2 (11.6–12.8)	6.0 (5.8–6.2)	4.6 (4.4–4.8)	44.0 (42–46)	23.0 (22–24)	8.1 (7.8–8.4)	0.82 (0.79–0.84)	0.09 (0.08–0.09)
GH, GH2, GH3, GH4, PH, PH2, PH3, PH4	24	110.4 (88.6–126.2)	48.8 (39.2–56.4)	24.8 (20.4–28.2)	21.2 (16.5–25.0)	131.6 (94–168)	76.8 (54–97)	34.3 (20.8–46.1)	2.89 (2.10–3.71)	0.59 (0.34–0.91)
GB, GR, PB, PR	12	78.9 (74.6–83.5)	35.0 (33.1–37.2)	18.0 (17.0–19.1)	12.5 (11.4–13.7)	128.3 (119–139)	87.5 (78–97)	35.0 (31.5–38.8)	2.91 (2.68–3.18)	0.64 (0.56–0.73)
GW, PW, PFW	9	65.1 (61.5–69.8)	27.4 (25.7–29.2)	13.9 (12.9–15.0)	9.5 (8.6–10.4)	106.0 (101–111)	67.7 (63–74)	26.6 (24.6–28.7)	2.35 (2.22–2.48)	0.45 (0.42–0.49)
PRC	3	92.6	41.6	21.0	16.8	149	92	45.6	3.40	0.74
GHB, PHB	6	118.1 (117.4–118.8)	51.9 (51.0–52.8)	26.2 (25.8–26.5)	23.2 (22.1–24.2)	145.0 (144–146)	89.0 (88–90)	44.5 (44.2–44.8)	3.63 (3.55–3.70)	0.73 (0.70–0.76)

Note: “Analyzed samples (*n*)” refers to the number of independently prepared experimental beverage samples included in each processing group. Each experimental variant was represented by three independently prepared samples (*n* = 3). For each independent sample, triplicate instrumental readings were performed and averaged before statistical analysis; these instrumental readings were not treated as independent samples. Values are reported as the arithmetic mean of the variant-level means included in each row, with the minimum and maximum variant-level means shown in parentheses. The broader wood-associated group comprises GW, PW, and PFW, whereas GHB and PHB represent the mixed honey–blackthorn variants. PRC is presented separately as a single intensified rosehip-macerated variant; therefore, only its variant-level mean is shown. Detailed mean ± SD values for each individual experimental variant are provided in the Supplementary Materials.

**Table 3:** Concentrations of trace elements potentially influenced by additives, post-distillation processing, and contact materials, together with potentially toxic elements, in the analyzed Pălincă-based beverage groups and the number of independent samples included in each processing category (total *n* = 60 independent samples).

Sample Codes	Analyzed Samples (*n*)	Al µg/L	Fe µg/L	Cu µg/L	Zn µg/L	Mn µg/L	Co µg/L	Se µg/L	Ba µg/L	Pb µg/L	Cd µg/L	As µg/L	Hg µg/L	Ni µg/L	Cr µg/L
GS, PS	6	36.0 (34–38)	109.0 (106–112)	280.0 (276–284)	71.0 (68–74)	17.0 (16–18)	0.41 (0.40–0.42)	1.7 (1.6–1.8)	23.0 (22–24)	11.5 (11–12)	1.15 (1.1–1.2)	3.0 (3.0–3.1)	0.17 (0.17–0.18)	6.2 (6.1–6.4)	4.1 (4.0–4.2)
GH, GH2, GH3, GH4, PH, PH2, PH3, PH4	24	119.4 (82–176)	462.2 (248–852)	382.2 (336–458)	219.8 (156–326)	87.8 (52–152)	1.35 (0.82–2.28)	4.8 (3.0–7.6)	79.2 (54–116)	16.6 (14–22)	1.77 (1.5–2.3)	5.3 (4.2–7.0)	0.27 (0.22–0.37)	11.8 (8.6–18.2)	7.3 (5.6–10.6)
GB, GR, PB, PR	12	126.8 (118–136)	584.5 (548–624)	415.0 (398–432)	231.0 (216–246)	105.5 (96–116)	1.58 (1.48–1.68)	5.3 (4.8–5.8)	79.5 (74–86)	17.0 (16–18)	1.75 (1.7–1.8)	5.0 (4.8–5.3)	0.27 (0.25–0.28)	12.2 (11.6–12.8)	6.9 (6.6–7.2)
GW, PW, PFW	9	103.3 (96–112)	433.3 (412–462)	504.7 (488–524)	189.3 (182–198)	80.0 (74–86)	1.19 (1.12–1.26)	4.4 (4.2–4.6)	66.0 (62–70)	19.7 (19–21)	2.0 (1.9–2.2)	5.9 (5.4–6.6)	0.32 (0.30–0.35)	15.0 (13.8–16.8)	8.9 (8.2–10.0)
PRC	3	154.0	706.0	438.0	278.0	128.0	1.92	6.4	96.0	20.0	2.0	6.0	0.32	15.6	9.0
GHB, PHB	6	164.0 (162–166)	770.0 (744–796)	462.0 (452–472)	309.0 (304–314)	140.0 (138–142)	2.04 (2.02–2.06)	6.9 (6.8–7.0)	107.0 (104–110)	19.5 (19–20)	2.05 (2.0–2.1)	6.2 (6.1–6.2)	0.33 (0.30–0.36)	16.2 (16.0–16.4)	9.5 (9.4–9.6)

Note: “Analyzed samples (*n*)” refers to the number of independently prepared experimental beverage samples included in each processing group. Each experimental variant was represented by three independently prepared samples (*n* = 3). For each independent sample, triplicate instrumental readings were performed and averaged before statistical analysis; these instrumental readings were not treated as independent samples. Except for PRC, values are reported as the arithmetic mean of the variant-level means included in each row, with the minimum and maximum variant-level means shown in parentheses. The broader wood-associated group comprises GW, PW, and PFW, whereas GHB and PHB represent the mixed honey–blackthorn variants. PRC is presented separately as a single intensified rosehip-macerated variant; therefore, only its variant-level mean is shown. Detailed mean ± SD values for each individual experimental variant are provided in the Supplementary Materials.

**Table 4:** Summary of non-carcinogenic risk characterization of the experimental Romanian artisanal Pălincă-based beverages based on THQ and HI values.

Technological Category	Analyzed Samples (*n*)	HI Range	Mean HI	Maximum Individual THQ Range	Dominant THQ Contributors	Sample with Highest HI	Elemental Risk Interpretation
Traditional control Pălincă distillates	6	0.0129–0.0137	0.0133	0.00518–0.00533	Cu, Fe, Zn	GS	Low non-carcinogenic elemental risk
Honey-enriched grape Pălincă-based beverages	12	0.0192–0.0297	0.0241	0.00630–0.00784	Cu, Fe, Zn, Mn	GH4	
Honey-enriched plum Pălincă-based beverages	12	0.0198–0.0356	0.0262	0.00645–0.00859	Cu, Fe, Zn, Mn	PH4	
Fruit-macerated Pălincă-based beverages	12	0.0256–0.0285	0.0271	0.00746–0.00810	Cu, Fe, Zn	PB	
Wood-associated Pălincă-based beverages	9	0.0265–0.0297	0.0279	0.00915–0.00983	Fe, Cu, Zn, Mn	PFW	
Mixed/intensified botanical Pălincă-based beverages	9	0.0315–0.0333	0.0325	0.00821–0.00885	Fe, Cu, Mn, Zn, Co, Pb	GHB	

Note: “Analyzed samples (*n*)” refers to independent analyzed samples included in each technological category. Each experimental variant was represented by three independent samples, and each sample was measured using three instrumental replicate readings. Instrumental readings were averaged before statistical analysis and were not treated as independent samples. The wood-associated category included GW, PW, and PFW, whereas the mixed/intensified botanical category included PRC, GHB, and PHB. HI values below 1.0 indicate low non-carcinogenic elemental risk under the applied exposure scenario.

**Table 5:** Hierarchical cluster analysis (HCA)-derived grouping of the experimental Pălincă-based beverage variants and their principal treatment-related characteristics.

HCA Cluster	Variants Included	Technological Interpretation/Exact Variant Definition	Main Treatment-Related Characteristics
I	GS, PS	Control distillates: GS = traditional grape Pălincă distillate; PS = traditional plum Pălincă distillate	Low conductivity; low TPC and DPPH; low mineral content; low turbidity
II	PH, PH2, PH3, PH4, GH, GH2, GH3, GH4	Honey-enriched variants: grape- and plum-based Pălincă beverages enriched with honey at 1/3, 1/2, 2/3, and 3/4 (*v*/*v*)	High conductivity; increased K, Rb, and Ba; higher TPC and DPPH; increased dry matter and turbidity
III	GB, PB, GR, PR, PRC	Fruit-associated cluster: GB and PB = blackthorn-macerated grape and plum Pălincă-based beverages; GR and PR = rosehip-macerated grape and plum Pălincă-based beverages; PRC = plum Pălincă-based beverage obtained through intensified maceration with fragmented rosehips	High TPC and DPPH; increased a* and b* values; enriched in phenolic compounds; distinct color intensity
IV	GW, PW, PFW	Wood-associated maturation variants: GW = grape Pălincă matured with plum wood fragments; PW = plum Pălincă matured with plum wood fragments; PFW = plum Pălincă matured exclusively with mulberry wood fragments, without added fruit material	Higher Cu and Fe; moderate TPC; maturation-associated color changes
V	GHB, PHB	Mixed honey–botanical variants: GHB = grape Pălincă + honey + blackthorn; PHB = plum Pălincă + honey + blackthorn	Very high conductivity; highest TPC and DPPH; high mineral content; high turbidity

Note: The five clusters represent empirical groupings obtained by hierarchical cluster analysis and are not fully identical to the a priori technological categories. PRC was prepared from plum Pălincă through intensified maceration with fragmented rosehips and contained neither cinnamon nor blackthorn fruits. Although PRC was classified a priori as an intensified botanical variant, it grouped with the fruit-macerated variants because of its rosehip-associated compositional profile. PFW was prepared exclusively by maturation of plum Pălincă with mulberry wood fragments, without the addition of fruit material, and was classified as a wood-associated maturation variant. All honey levels refer to the volume fraction (*v*/*v*) of honey in the final beverage. The analysis was based on 20 variant-level means, each calculated from three independently prepared replicate beverage samples.

**Table 6:** Integrated technological approaches, treatment-related contributions, and observed outcomes of the experimental Pălincă-based beverage variants.

Section	Technological Approach/Analytical Domain	Experimental Variants or Treatment	Main Associated Constituents/Parameters	Main Observed Outcomes
Technological approaches	Honey enrichment	GH–GH4 and PH–PH4; progressive honey incorporation at 1/3, 1/2, 2/3, and 3/4 *v*/*v*	Sugars, organic acids, phenolic compounds, and minerals	Decreased alcoholic strength; increased °Brix, density, conductivity, turbidity, total phenolic content, DPPH activity, and mineral content; decreased L* and increased b*
Technological approaches	Fruit maceration	GB and PB: blackthorn fruits; GR and PR: rosehips	Anthocyanins, carotenoids, phenolic acids, tannins, and other plant-derived constituents	Increased total phenolic content and DPPH activity; stronger chromatic differentiation, particularly increased redness and/or yellowness; broader treatment-related compositional profiles
Technological approaches	Wood-assisted maturation	GW: grape Pălincă matured with plum wood fragments; PW: plum Pălincă matured with plum wood fragments; PFW: plum Pălincă matured with mulberry wood fragments	Wood-associated phenolic constituents, organic extractives, minerals, and trace elements	Moderate increases in phenolic content and DPPH activity; decreased L* and increased b*; treatment-related elemental and chromatic changes associated with wood contact
Technological approaches	Mixed honey–botanical variants	GHB: grape Pălincă with honey and blackthorn fruits; PHB: plum Pălincă with honey and blackthorn fruits	Combined honey- and blackthorn-associated constituents	Higher measured TPC and DPPH responses; increased turbidity and mineral content; broader chromatic and elemental variation
Technological approaches	Intensified botanical variant	PRC: plum Pălincă subjected to intensified maceration with fragmented rosehips	Rosehip-derived pigments, phenolic compounds, carotenoid-associated constituents, and minerals	Enhanced extraction associated with the increased contact surface; stronger chromatic, phenolic, antioxidant, and elemental responses than the simple rosehip-macerated variant
Observed effects	Physicochemical transformation	All experimental variants	Alcoholic strength, °Brix, density, electrical conductivity, and turbidity	Alcoholic strength decreased after enrichment, whereas °Brix, density, conductivity, and turbidity generally increased
Observed effects	Phenolic and DPPH response	All experimental variants	Total phenolic content and DPPH radical-scavenging activity	Higher responses were observed particularly in honey-enriched, fruit-macerated, mixed, and intensified variants
Observed effects	Chromatic differentiation	All experimental variants	L*, a*, and b* coordinates	L* generally decreased, while a* and/or b* increased depending on the botanical material and processing treatment
Observed effects	Elemental profile and differentiation	All experimental variants	K, Mg, Sr, Rb, Li, Cs, Ba, Fe, Cu, Zn, Mn, and other quantified elements	Treatment-related elemental differentiation was observed among the experimental beverages
Observed effects	Screening-level non-carcinogenic exposure assessment	Assessed elemental fraction under the stated exposure scenario	Individual THQ values and cumulative HI values	All THQ and HI values remained below 1 under the stated exposure assumptions
Classification clarification	PFW and PRC	PFW contained mulberry wood fragments only; PRC contained fragmented rosehips only	PFW contained no fruit material; PRC contained neither cinnamon nor blackthorn fruits	PFW is classified exclusively as a wood-associated maturation variant, whereas PRC is classified as an intensified rosehip-macerated plum variant

Note: GH–GH4 and PH–PH4 represent grape- and plum Pălincă-based beverages enriched with honey at 1/3, 1/2, 2/3, and 3/4 *v*/*v*, respectively. GB and PB were macerated with blackthorn fruits, whereas GR and PR were macerated with rosehips. GW and PW were matured with plum wood fragments. PFW was prepared exclusively from plum Pălincă matured with mulberry wood fragments, without the addition of fruit material, and was classified as a wood-associated maturation variant. GHB and PHB contained honey and blackthorn fruits. PRC was prepared by intensified maceration of plum Pălincă with fragmented rosehips and contained neither cinnamon nor blackthorn fruits. TPC, total phenolic content; DPPH, 2,2-diphenyl-1-picrylhydrazyl radical-scavenging activity; THQ, target hazard quotient; HI, hazard index. Each experimental variant was represented by three independently prepared beverage samples (*n* = 3).

**Table 7:** Technological classification, composition, and experimental coding of the investigated artisanal Pălincă-based beverage variants.

Technological Category	Code	Base Matrix	Additive or Treatment	Exact Experimental Description	Independent Samples (*n*)
Control distillates	GS	Grape Pălincă	None	Traditional grape Pălincă distillate used as control	3
Control distillates	PS	Plum Pălincă	None	Traditional plum Pălincă distillate used as control	3
Honey-enriched variants	GH	Grape Pălincă	Honey, 1/3 *v*/*v*	Grape Pălincă-based beverage enriched with honey at 1/3 *v*/*v*	3
Honey-enriched variants	GH2	Grape Pălincă	Honey, 1/2 *v*/*v*	Grape Pălincă-based beverage enriched with honey at 1/2 *v*/*v*	3
Honey-enriched variants	GH3	Grape Pălincă	Honey, 2/3 *v*/*v*	Grape Pălincă-based beverage enriched with honey at 2/3 *v*/*v*	3
Honey-enriched variants	GH4	Grape Pălincă	Honey, 3/4 *v*/*v*	Grape Pălincă-based beverage enriched with honey at 3/4 *v*/*v*	3
Honey-enriched variants	PH	Plum Pălincă	Honey, 1/3 *v*/*v*	Plum Pălincă-based beverage enriched with honey at 1/3 *v*/*v*	3
Honey-enriched variants	PH2	Plum Pălincă	Honey, 1/2 *v*/*v*	Plum Pălincă-based beverage enriched with honey at 1/2 *v*/*v*	3
Honey-enriched variants	PH3	Plum Pălincă	Honey, 2/3 *v*/*v*	Plum Pălincă-based beverage enriched with honey at 2/3 *v*/*v*	3
Honey-enriched variants	PH4	Plum Pălincă	Honey, 3/4 *v*/*v*	Plum Pălincă-based beverage enriched with honey at 3/4 *v*/*v*	3
Simple fruit-macerated variants	GB	Grape Pălincă	Blackthorn fruits	Grape Pălincă-based beverage macerated with blackthorn fruits	3
Simple fruit-macerated variants	GR	Grape Pălincă	Rosehips	Grape Pălincă-based beverage macerated with rosehips	3
Simple fruit-macerated variants	PB	Plum Pălincă	Blackthorn fruits	Plum Pălincă-based beverage macerated with blackthorn fruits	3
Simple fruit-macerated variants	PR	Plum Pălincă	Rosehips	Plum Pălincă-based beverage macerated with rosehips	3
Wood-associated variants	GW	Grape Pălincă	Plum wood fragments	Grape Pălincă-based beverage matured with plum wood fragments	3
Wood-associated variants	PW	Plum Pălincă	Plum wood fragments	Plum Pălincă-based beverage matured with plum wood fragments	3
Wood-associated variants	PFW	Plum Pălincă	Mulberry wood fragments	Plum Pălincă-based beverage matured with mulberry wood fragments; no fruit material was added during maturation	3
Mixed/intensified botanical variants	PRC	Plum Pălincă	Fragmented rosehips	Plum Pălincă-based beverage obtained through intensified maceration with fragmented rosehips	3
Mixed/intensified botanical variants	GHB	Grape Pălincă	Honey and blackthorn fruits	Mixed grape Pălincă-based beverage with honey and blackthorn fruits	3
Mixed/intensified botanical variants	PHB	Plum Pălincă	Honey and blackthorn fruits	Mixed plum Pălincă-based beverage with honey and blackthorn fruits	3

Note: The experimental design comprised 20 variants, each represented by three independently prepared beverage samples, resulting in 60 independent experimental samples. For each sample, three instrumental replicate readings were obtained and averaged before statistical analysis. Instrumental readings were not treated as independent experimental units. PFW was prepared exclusively by maturation of plum Pălincă with mulberry wood fragments, without the addition of rosehips, blackthorn fruits, or any other fruit material. PRC was prepared through intensified maceration of plum Pălincă with fragmented rosehips and did not contain cinnamon or blackthorn fruits.

## Data Availability

The data presented in this study are available in the article and Supplementary Materials. Additional raw data supporting the findings of this study are available from the corresponding author upon reasonable request.

## Supplementary Materials

The following supporting information can be downloaded at: https://www.mdpi.com/article/10.3390/molecules31152673/s1, Table S1: Physicochemical, chromatic, and functional characterization parameters determined for the experimental Romanian artisanal Pălincă-based beverages produced in the Tinca area (Bihor County, north-western Romania); Table S2: Major mineral composition and process-related elemental markers determined in the experimental Romanian artisanal Pălincă-based beverages produced in the Tinca area (Bihor County, north-western Romania) using ICP-MS analysis; Table S3: Process-, additive-, and contact-related trace elements and potentially toxic elements determined in the experimental Romanian artisanal Pălincă-based beverages produced in the Tinca area (Bihor County, north-western Romania) using ICP-MS analysis; Table S4: Dietary exposure assessment based on estimated daily intake (EDI) and relative exposure increase calculated for the investigated experimental Romanian artisanal Pălincă-based beverages; Table S5: Integrated THQ- and HI-based non-carcinogenic elemental risk evaluation and comparative risk characterization of experimental Romanian artisanal Pălincă-based beverages; Table S6: Integrated physicochemical, chromatic, functional, and elemental modifications induced by progressive honey enrichment in the experimental Romanian artisanal Pălincă-based beverages; Table S7: Integrated overview of the principal chromatic, functional, physicochemical, and elemental modifications induced by fruit maceration in the experimental Romanian artisanal Pălincă-based beverages; Table S8: Integrated overview of the principal physicochemical, oxidative, chromatic, functional, and elemental modifications induced by wood-assisted maturation in the experimental Romanian artisanal Pălincă-based beverages; Table S9: Integrated overview of the principal ICP-MS elemental profiling patterns and technological discrimination features identified in the investigated Romanian artisanal Pălincă-based beverages; Table S10: Integrated overview of the principal technological trace elements, contamination pathways, and processing-associated elemental modifications identified in the investigated Romanian artisanal Pălincă-based beverages; Table S11: Integrated overview of the principal potentially toxic elements, technological accumulation patterns, and elemental safety and non-carcinogenic risk assessment parameters identified in the investigated Romanian artisanal Pălincă-based beverages; Table S12: Integrated overview of the principal non-carcinogenic human health risk assessment parameters, exposure characteristics, and toxicological safety indicators identified in the investigated Romanian artisanal Pălincă-based beverages based on THQ and HI evaluation models; Table S13: Integrated overview of the principal correlation patterns and chemometric differentiation features identified among the experimental Romanian artisanal Pălincă-based beverages; Table S14: Integrated overview of the principal technological significance, process-related differentiation potential, and functional valorization characteristics identified in the newly developed Romanian artisanal Pălincă-based beverages; Figure S1: Supplementary process map of the experimental design, sample processing, quality-control checkpoints, contamination-prevention strategy, traceability records, and analytical allocation used for the investigated Romanian artisanal Pălincă-based beverages; Table S15: Experimental classification, sample coding, and technological categories of the investigated artisanal Pălincă-based beverages; Table S16: Standardized additive proportions and maceration/maturation periods applied for the preparation of the experimental artisanal Pălincă-based beverages produced in the Tinca area, Bihor County, north-western Romania; Table S17: Pre-treatment procedures and fragmentation characteristics of the botanical and maturation materials used for the preparation of the experimental artisanal Pălincă-based beverages; Table S18: Comprehensive ICP-MS analytical validation, calibration performance, sensitivity parameters, precision, uncertainty estimation, and quality assurance criteria applied for the multi-elemental characterization of the investigated Romanian artisanal Pălincă-based beverages; Table S19: Microwave-assisted digestion program, thermal mineralization stages, and operational pressure conditions applied prior to ICP-MS multi-elemental analysis of the experimental artisanal Pălincă-based beverages; Table S20: ICP-MS analytical sequence design, quality control verification frequency, procedural blank monitoring, and sample randomization strategy applied during the multi-elemental analysis of the experimental artisanal Pălincă-based beverages; Table S21: Detailed ICP-MS instrumental operating conditions, acquisition parameters, optimization criteria, and analytical sequence settings applied for the multi-elemental characterization of the investigated Romanian artisanal Pălincă-based beverages; Table S22: Certified reference material and certified reference solution verification data, certified concentrations, experimentally determined concentrations, recovery efficiency, and analytical trueness parameters applied for the ICP-MS multi-elemental characterization of the investigated Romanian artisanal Pălincă-based beverages.

## Abbreviations

The following abbreviations are used in this manuscript:

ANOVA	Analysis of variance
Ar	Argon gas
AT	Averaging time
BEC	Background equivalent concentration
BW	Body weight
C	Concentration of the investigated element in the analyzed beverage
CDI	Chronic Daily Intake
CIELAB	Commission Internationale de l’Éclairage L*a*b* color system
CRM	Certified reference material
CV	Coefficient of variation
DI	Daily intake rate
DPPH	2,2-diphenyl-1-picrylhydrazyl radical scavenging activity
ED	Exposure duration
EDI	Estimated Daily Intake
EF	Exposure frequency
EPA	United States Environmental Protection Agency
EU	European Union
GAE	Gallic acid equivalents
GI	Geographical Indication for spirit drinks
HCA	Hierarchical cluster analysis
He-KED	Helium kinetic energy discrimination mode
HI	Hazard Index
ICP-MS	Inductively coupled plasma mass spectrometry
L*	Lightness parameter
a*	Red-green chromatic coordinate
b*	Yellow-blue chromatic coordinate
LoD	Limit of detection
LoQ	Limit of quantification
n	Number of replicate determinations or samples
NTU	Nephelometric turbidity units
PC1	First principal component
PC2	Second principal component
PCA	Principal component analysis
PTFE	Polytetrafluoroethylene
QA/QC	Quality assurance and quality control
QC	Quality control verification standard
R^2^	Coefficient of determination
RF	Radiofrequency
RfD	Oral reference dose
RSD	Relative standard deviation
SD	Standard deviation
SPSS	Statistical Package for the Social Sciences
TPC	Total phenolic content
THQ	Target Hazard Quotient
TTHQ	Total Target Hazard Quotient
UV–Vis	Ultraviolet–visible spectrophotometry
*v*/*v*	Volume/volume ratio
WHO	World Health Organization
GS	Traditional grape Pălincă distillate
PS	Traditional plum Pălincă distillate
GH	Experimental grape Pălincă-based beverage with honey, 1/3 honey, *v*/*v*
GH2	Experimental grape Pălincă-based beverage with honey, 1/2 honey, *v*/*v*
GH3	Experimental grape Pălincă-based beverage with honey, 2/3 honey, *v*/*v*
GH4	Experimental grape Pălincă-based beverage with honey, 3/4 honey, *v*/*v*
PH	Experimental plum Pălincă-based beverage with honey, 1/3 honey, *v*/*v*
PH2	Experimental plum Pălincă-based beverage with honey, 1/2 honey, *v*/*v*
PH3	Experimental plum Pălincă-based beverage with honey, 2/3 honey, *v*/*v*
PH4	Experimental plum Pălincă-based beverage with honey, 3/4 honey, *v*/*v*
GB	Experimental grape Pălincă-based beverage macerated with blackthorn fruits
GR	Experimental grape Pălincă-based beverage macerated with rosehips
GW	Experimental grape Pălincă-based beverage matured with plum wood
GHB	Mixed grape Pălincă-based beverage with honey and blackthorn fruits
PB	Experimental plum Pălincă-based beverage macerated with blackthorn fruits
PR	Experimental plum Pălincă-based beverage macerated with rosehips
PW	Experimental plum Pălincă-based beverage matured with plum wood
PRC	Experimental plum Pălincă-based beverage with fragmented rosehips
PFW	Experimental plum Pălincă-based beverage matured with mulberry wood
PHB	Mixed plum Pălincă-based beverage with honey and blackthorn fruits
