# Supplementary material for: ICP-MS Elemental Profiling and Antioxidant Characterization of Romanian Artisanal Pălincă-Based Beverages Enriched with Honey, Wild Fruits, and Wood Materials
Source: Molecules. 2026 Jul 31;31(15):2673. doi: 10.3390/molecules31152673 (PMC13468524; doi:10.3390/molecules31152673)
Supplement: Supplementary file 1 [file molecules-31-02673-s001.zip › molecules-4426083-supplementary.pdf]

molecules

MDPI

Table S1. Physicochemical, chromatic, technological, and functional characterization parameters determined for the experimental Romanian artisanal Pălincă-based beverages produced in the Tinca area (Bihor County, north-western Romania).

Sample Code	Alcoholic Strength (% v/v)	pH	Electrical Conductivity (μS/cm)	Density (g/cm³)	Turbidity (NTU)	Total Soluble Solids (°Brix)	L*	a*	b*	Dry Matter (g/L)	Total Phenolic Content (mg GAE/L)	DPPH Inhibition (%)
GS	52.4 ± 0.6^{b}	4.28 ± 0.04^{a}	186 ± 11^{j}	0.934 ± 0.002^{j}	1.8 ± 0.2^{h}	1.4 ± 0.1^{h}	96.2 ± 1.1^{a}	0.8 ± 0.1^{h}	2.4 ± 0.2^{j}	1.9 ± 0.2^{j}	42 ± 5^{j}	18.4 ± 1.7^{j}
GH	46.8 ± 0.5^{f}	4.11 ± 0.05^{c}	412 ± 24^{f}	0.961 ± 0.003^{f}	6.4 ± 0.5^{f}	10.6 ± 0.7^{d}	82.5 ± 1.8^{b}	-2.1 ± 0.2^{j}	18.4 ± 1.3^{h}	12.8 ± 0.9^{e}	186 ± 12^{h}	46.8 ± 3.5^{h}
GH2	45.6 ± 0.4^{h}	4.05 ± 0.04^{d}	438 ± 26^{e}	0.966 ± 0.003^{e}	7.2 ± 0.5^{e}	11.8 ± 0.8^{c}	79.6 ± 1.9^{c}	-1.6 ± 0.2^{i}	20.8 ± 1.5^{g}	13.9 ± 1.0^{d}	228 ± 16^{g}	52.7 ± 3.8^{g}
GH3	44.8 ± 0.4^{i}	3.99 ± 0.04^{e}	462 ± 27^{c}	0.969 ± 0.003^{c}	8.6 ± 0.6^{c}	13.1 ± 0.9^{b}	74.2 ± 2.1^{d}	-1.2 ± 0.1^{hi}	22.9 ± 1.6^{f}	15.2 ± 1.1^{c}	264 ± 18^{f}	58.4 ± 4.1^{f}
GH4	44.1 ± 0.3^{j}	3.93 ± 0.04^{f}	488 ± 28^{b}	0.972 ± 0.004^{b}	10.1 ± 0.7^{a}	14.4 ± 1.0^{a}	69.5 ± 2.4^{e}	-0.8 ± 0.1^{h}	25.7 ± 1.8^{e}	16.7 ± 1.2^{b}	304 ± 21^{e}	62.8 ± 4.4^{e}
GB	49.2 ± 0.7^{d}	3.96 ± 0.03^{e}	368 ± 18^{h}	0.948 ± 0.002^{h}	8.2 ± 0.6^{d}	6.2 ± 0.4^{f}	54.6 ± 2.5^{i}	28.4 ± 1.7^{b}	8.3 ± 0.6^{h}	9.4 ± 0.7^{h}	472 ± 30^{c}	70.2 ± 4.7^{c}
GR	48.6 ± 0.6^{de}	3.82 ± 0.04^{h}	342 ± 16^{i}	0.946 ± 0.003^{i}	7.1 ± 0.5^{e}	5.8 ± 0.3^{f}	68.2 ± 2.0^{f}	14.6 ± 1.1^{f}	32.5 ± 2.3^{b}	8.8 ± 0.6^{h}	438 ± 28^{d}	68.7 ± 4.3^{d}
GW	50.8 ± 0.5^{c}	4.19 ± 0.03^{b}	296 ± 14^{h}	0.941 ± 0.002^{j}	3.2 ± 0.3^{g}	3.6 ± 0.2^{g}	61.5 ± 1.9^{h}	9.6 ± 0.7^{g}	24.7 ± 1.6^{f}	6.7 ± 0.5^{i}	296 ± 22^{f}	52.1 ± 3.9^{g}
GHB	45.9 ± 0.4^{g}	3.88 ± 0.05^{g}	486 ± 28^{b}	0.971 ± 0.004^{b}	10.2 ± 0.8^{a}	12.4 ± 0.9^{c}	48.4 ± 2.8^{j}	22.5 ± 1.6^{d}	10.4 ± 0.7^{g}	14.9 ± 1.1^{c}	596 ± 37^{a}	78.1 ± 5.0^{a}
PS	53.6 ± 0.6^{a}	4.34 ± 0.04^{a}	194 ± 12^{j}	0.932 ± 0.002^{j}	1.7 ± 0.2^{h}	1.2 ± 0.1^{h}	97.1 ± 1.0^{a}	0.5 ± 0.1^{h}	2.1 ± 0.2^{j}	1.7 ± 0.2^{j}	38 ± 4^{j}	16.2 ± 1.4^{j}
PH	47.1 ± 0.5^{f}	4.09 ± 0.04^{c}	428 ± 25^{f}	0.964 ± 0.003^{f}	6.8 ± 0.5^{f}	11.2 ± 0.8^{d}	84.1 ± 1.7^{b}	-1.8 ± 0.2^{j}	19.6 ± 1.4^{h}	13.2 ± 0.9^{d}	194 ± 14^{h}	48.6 ± 3.6^{h}
PH2	45.9 ± 0.4^{g}	4.02 ± 0.04^{d}	446 ± 26^{d}	0.968 ± 0.003^{d}	7.5 ± 0.5^{e}	12.1 ± 0.8^{c}	80.4 ± 1.8^{c}	-1.4 ± 0.2^{i}	21.3 ± 1.5^{g}	14.2 ± 1.0^{d}	236 ± 17^{g}	54.1 ± 3.9^{g}
PH3	45.1 ± 0.4^{h}	3.96 ± 0.04^{e}	471 ± 28^{bc}	0.970 ± 0.004^{c}	8.9 ± 0.6^{c}	13.6 ± 0.9^{b}	75.1 ± 2.0^{d}	-1.0 ± 0.1^{hi}	23.8 ± 1.7^{f}	15.8 ± 1.1^{b}	286 ± 20^{f}	61.9 ± 4.4^{f}
PH4	44.3 ± 0.3^{j}	3.89 ± 0.04^{g}	502 ± 30^{a}	0.973 ± 0.004^{a}	10.4 ± 0.8^{a}	14.8 ± 1.0^{a}	70.2 ± 2.3^{e}	-0.6 ± 0.1^{h}	26.4 ± 1.9^{e}	17.1 ± 1.2^{a}	338 ± 24^{e}	68.2 ± 4.8^{e}
PB	49.4 ± 0.6^{d}	3.92 ± 0.03^{f}	376 ± 19^{g}	0.949 ± 0.003^{h}	8.5 ± 0.6^{c}	6.5 ± 0.4^{f}	52.8 ± 2.4^{i}	29.1 ± 1.8^{a}	8.7 ± 0.6^{h}	9.8 ± 0.7^{g}	512 ± 35^{b}	74.4 ± 5.0^{b}
PR	48.9 ± 0.5^{e}	3.79 ± 0.04^{i}	348 ± 17^{i}	0.947 ± 0.003^{i}	7.3 ± 0.5^{e}	5.9 ± 0.4^{f}	66.7 ± 2.1^{f}	15.2 ± 1.2^{f}	33.6 ± 2.4^{ab}	9.0 ± 0.6^{h}	452 ± 29^{cd}	69.8 ± 4.4^{c}
PW	51.2 ± 0.5^{c}	4.16 ± 0.03^{b}	304 ± 15^{h}	0.942 ± 0.002^{j}	3.5 ± 0.3^{g}	3.8 ± 0.2^{g}	59.8 ± 1.8^{h}	10.1 ± 0.8^{g}	25.4 ± 1.7^{e}	6.9 ± 0.5^{i}	322 ± 24^{e}	55.3 ± 4.0^{g}
PRC	47.8 ± 0.5^{f}	3.72 ± 0.04^{j}	396 ± 21^{f}	0.953 ± 0.003^{g}	9.8 ± 0.7^{b}	7.4 ± 0.5^{e}	62.4 ± 2.3^{h}	17.4 ± 1.3^{e}	38.2 ± 2.6^{a}	10.8 ± 0.8^{f}	556 ± 37^{b}	77.4 ± 5.3^{b}
PFW	50.6 ± 0.5^{c}	4.14 ± 0.03^{b}	318 ± 16^{h}	0.944 ± 0.002^{i}	3.9 ± 0.3^{g}	4.1 ± 0.3^{g}	55.3 ± 1.9^{i}	12.4 ± 0.9^{g}	28.1 ± 1.9^{d}	7.5 ± 0.6^{i}	346 ± 26^{e}	59.1 ± 4.2^{f}
PHB	46.2 ± 0.4^{g}	3.84 ± 0.05^{h}	498 ± 29^{a}	0.972 ± 0.004^{a}	10.6 ± 0.8^{a}	12.9 ± 0.9^{b}	46.8 ± 2.9^{j}	23.8 ± 1.7^{c}	11.2 ± 0.8^{g}	15.3 ± 1.2^{b}	648 ± 42^{a}	82.4 ± 5.5^{a}

F-value	82.6	40.2	296.8	146.2	191.8	264.4	178.5	392.1	231.4	208.6	438.2	326.4
p-value	<0.001	<0.001	<0.001	<0.001	<0.001	<0.001	<0.001	<0.001	<0.001	<0.001	<0.001	<0.001
CV (%)	5.71	4.06	23.84	1.32	39.72	51.38	20.41	n.a.*	48.96	40.88	44.15	27.63
η^2	0.78	0.69	0.86	0.81	0.84	0.87	0.82	0.89	0.85	0.84	0.89	0.87
Range (min–max)	44.1–53.6	3.72–4.34	186–502	0.932–0.973	1.7–10.6	1.2–14.8	46.8–97.1	−2.1–29.1	2.1–38.2	1.7–17.1	38–648	16.2–82.4
Overall mean	47.92	4.00	388.54	0.956	7.11	8.67	69.01	8.72	20.41	11.12	338.4	59.4

Values are expressed as mean ± standard deviation (SD) of three independent samples per experimental variant (n = 3). Each independent sample was measured using triplicate instrumental readings, which were averaged before statistical analysis. Different superscript lowercase letters within the same column indicate statistically significant differences among experimental variants according to one-way analysis of variance (ANOVA) followed by Duncan's multiple range test ($p < 0.05$). Samples sharing at least one identical superscript letter are not significantly different, whereas samples marked with different letters differ significantly at the 95% confidence level. One-way ANOVA revealed highly significant effects of the applied technological treatments on all investigated physicochemical, chromatic, and functional parameters ($p < 0.001$).

Table S2. Major mineral composition and process-related elemental markers determined in the experimental Romanian artisanal Pălincă-based beverages produced in the Tinca area (Bihor County, north-western Romania) using ICP-MS analysis.

Sample Code	K (mg/L)	Ca (mg/L)	Mg (mg/L)	Na (mg/L)	Sr (µg/L)	Ba (µg/L)	Rb (µg/L)	Li (µg/L)	Cs (µg/L)
GS	28.4 ± 2.3^{h}	12.8 ± 1.1^{h}	6.2 ± 0.7^{h}	4.8 ± 0.5^{h}	46 ± 6^{h}	24 ± 4^{h}	8.4 ± 1.0^{h}	0.84 ± 0.12^{h}	0.09 ± 0.02^{h}
GH	88.6 ± 8.5^{f}	39.2 ± 4.0^{f}	20.4 ± 2.3^{f}	16.5 ± 2.1^{f}	94 ± 11^{f}	54 ± 8^{f}	20.8 ± 2.7^{f}	2.10 ± 0.28^{f}	0.34 ± 0.05^{f}
GH2	104.8 ± 10.2^{e}	45.8 ± 4.9^{e}	24.1 ± 2.7^{e}	19.8 ± 2.3^{e}	118 ± 14^{e}	69 ± 9^{e}	28.5 ± 3.5^{e}	2.86 ± 0.44^{e}	0.48 ± 0.07^{e}
GH3	116.2 ± 11.8^{c}	51.6 ± 5.5^{c}	25.7 ± 3.0^{c}	23.1 ± 2.7^{b}	142 ± 16^{c}	84 ± 11^{c}	39.2 ± 4.4^{c}	2.94 ± 0.39^{cd}	0.67 ± 0.10^{c}
GH4	121.5 ± 12.6^{b}	53.9 ± 5.9^{b}	27.1 ± 3.1^{b}	22.4 ± 2.6^{bc}	151 ± 18^{b}	91 ± 12^{b}	43.8 ± 5.0^{b}	3.32 ± 0.57^{bc}	0.74 ± 0.11^{b}
GB	79.8 ± 7.9^{g}	35.4 ± 3.8^{g}	18.2 ± 2.1^{g}	12.8 ± 1.7^{g}	132 ± 15^{d}	94 ± 12^{b}	36.4 ± 4.1^{c}	3.18 ± 0.63^{cd}	0.73 ± 0.09^{b}
GR	74.6 ± 7.2^{g}	33.1 ± 3.6^{g}	17.0 ± 1.9^{g}	11.4 ± 1.5^{g}	119 ± 13^{e}	78 ± 10^{d}	31.5 ± 3.6^{d}	2.74 ± 0.36^{de}	0.56 ± 0.08^{d}
GW	61.5 ± 5.9^{h}	25.7 ± 2.8^{h}	12.9 ± 1.5^{h}	8.6 ± 1.1^{h}	101 ± 12^{f}	63 ± 8^{e}	24.6 ± 2.9^{f}	2.22 ± 0.31^{ef}	0.42 ± 0.06^{f}
GHB	118.8 ± 12.1^{bc}	52.8 ± 5.7^{bc}	26.5 ± 3.0^{bc}	24.2 ± 2.8^{b}	146 ± 17^{bc}	88 ± 11^{bc}	44.8 ± 5.7^{b}	3.70 ± 0.71^{b}	0.76 ± 0.12^{b}
PS	26.8 ± 2.2^{h}	11.6 ± 1.0^{h}	5.8 ± 0.6^{h}	4.4 ± 0.5^{h}	42 ± 5^{h}	22 ± 3^{h}	7.8 ± 0.9^{h}	0.79 ± 0.10^{h}	0.08 ± 0.01^{h}
PH	96.4 ± 9.4^{f}	42.5 ± 4.5^{f}	21.8 ± 2.5^{f}	18.4 ± 2.2^{f}	97 ± 11^{f}	59 ± 8^{f}	23.4 ± 2.9^{f}	2.22 ± 0.34^{f}	0.39 ± 0.05^{f}
PH2	109.1 ± 10.7^{d}	47.8 ± 5.1^{d}	24.4 ± 2.8^{d}	20.7 ± 2.4^{d}	121 ± 14^{e}	71 ± 9^{e}	30.2 ± 3.6^{e}	2.76 ± 0.48^{e}	0.52 ± 0.08^{de}
PH3	120.7 ± 12.0^{b}	53.1 ± 5.8^{b}	26.9 ± 3.1^{b}	23.5 ± 2.7^{b}	162 ± 19^{a}	89 ± 11^{b}	42.6 ± 4.9^{b}	3.18 ± 0.43^{bc}	0.82 ± 0.13^{ab}
PH4	126.2 ± 13.1^{a}	56.4 ± 6.1^{a}	28.2 ± 3.2^{a}	25.0 ± 2.9^{a}	168 ± 20^{a}	97 ± 12^{ab}	46.1 ± 5.2^{a}	3.71 ± 0.66^{a}	0.91 ± 0.14^{a}
PB	83.5 ± 8.1^{g}	37.2 ± 4.0^{g}	19.1 ± 2.2^{g}	13.7 ± 1.8^{g}	139 ± 16^{c}	97 ± 13^{ab}	38.8 ± 4.5^{c}	3.05 ± 0.41^{d}	0.65 ± 0.10^{c}
PR	77.8 ± 7.5^{g}	34.4 ± 3.7^{g}	17.8 ± 2.0^{g}	12.1 ± 1.6^{g}	123 ± 14^{e}	81 ± 10^{cd}	33.2 ± 3.8^{d}	2.68 ± 0.37^{de}	0.60 ± 0.08^{cd}
PW	64.1 ± 6.2^{h}	27.4 ± 3.0^{h}	13.7 ± 1.6^{h}	9.5 ± 1.2^{h}	106 ± 12^{f}	66 ± 8^{e}	26.5 ± 3.1^{f}	2.34 ± 0.29^{e}	0.44 ± 0.06^{f}
PRC	92.6 ± 9.0^{f}	41.6 ± 4.4^{f}	21.0 ± 2.4^{f}	16.8 ± 2.0^{f}	149 ± 17^{b}	92 ± 12^{b}	45.6 ± 5.1^{ab}	3.40 ± 0.52^{bc}	0.74 ± 0.12^{b}
PFW	69.8 ± 6.8^{h}	29.2 ± 3.2^{h}	15.0 ± 1.7^{h}	10.4 ± 1.3^{h}	111 ± 13^{f}	74 ± 9^{d}	28.7 ± 3.3^{e}	2.48 ± 0.27^{e}	0.49 ± 0.07^{e}
PHB	117.4 ± 11.9^{c}	51.0 ± 5.5^{c}	25.8 ± 3.0^{c}	22.1 ± 2.6^{c}	144 ± 17^{bc}	90 ± 11^{b}	44.2 ± 5.1^{b}	3.55 ± 0.58^{bc}	0.70 ± 0.11^{bc}
F-value	30.84	28.12	24.10	35.76	19.35	16.84	24.92	14.92	17.66
p-value	<0.001	<0.001	<0.001	<0.001	<0.001	<0.001	<0.001	<0.001	<0.001

CV (%)	27.6	29.4	26.8	34.1	24.9	25.7	30.6	31.2	38.4
η^2	0.836	0.811	0.784	0.868	0.741	0.702	0.812	0.653	0.708
Range (min–max)	26.8–126.2	11.6–56.4	5.8–28.2	4.4–25.0	42–168	22–97	7.8–46.1	0.79–3.71	0.08–0.91
Overall mean	89.7	39.2	19.9	16.1	122.3	77.0	32.9	2.68	0.58

Values are expressed as mean ± standard deviation (SD) of three independent samples per experimental variant (n = 3). Each independent sample was measured using triplicate instrumental readings, which were averaged before statistical analysis. Different superscript lowercase letters within the same column indicate statistically significant differences among experimental variants according to one-way analysis of variance (ANOVA) followed by Duncan's multiple range test ($p < 0.05$). Samples sharing at least one identical superscript letter are not significantly different, whereas samples marked with different letters differ significantly at the 95% confidence level. One-way ANOVA revealed highly significant effects of the applied technological treatments on all investigated major mineral elements and process-related elemental markers ($p < 0.001$).

Table S3. Technological contamination indicators, trace process-related elemental markers, and potentially toxic elements determined in the experimental Romanian artisanal Pălincă-based beverages produced in the Tinca area (Bihor County, north-western Romania) using ICP-MS analysis.

Sample Code	Al (µg/L)	Fe (µg/L)	Cu (µg/L)	Zn (µg/L)	Mn (µg/L)	Co (µg/L)	Se (µg/L)	Ba (µg/L)	Pb (µg/L)	Cd (µg/L)	As (µg/L)	Hg (µg/L)	Ni (µg/L)	Cr (µg/L)
GS	38 ± 2.66^{h}	112 ± 7.84^{h}	284 ± 19.88^{h}	74 ± 5.18^{h}	18 ± 1.26^{h}	0.42 ± 0.03^{h}	1.8 ± 0.14^{h}	24 ± 1.68^{i}	12 ± 0.84^{j}	1.2 ± 0.10^{j}	3.1 ± 0.22^{j}	0.18 ± 0.01^{j}	6.4 ± 0.45^{j}	4.2 ± 0.29^{j}
GH	82 ± 5.74^{g}	248 ± 17.36^{g}	336 ± 23.52^{g}	156 ± 10.92^{g}	52 ± 3.64^{g}	0.82 ± 0.07^{g}	3.0 ± 0.24^{g}	54 ± 3.78^{h}	14 ± 0.98^{i}	1.5 ± 0.12^{i}	4.2 ± 0.29^{i}	0.22 ± 0.02^{i}	8.6 ± 0.60^{i}	5.6 ± 0.39^{i}
GH2	101 ± 7.07^{f}	332 ± 23.24^{f}	358 ± 25.06^{f}	184 ± 12.88^{f}	66 ± 4.62^{f}	1.04 ± 0.08^{f}	3.8 ± 0.27^{f}	64 ± 4.48^{fg}	15 ± 1.05^{h}	1.6 ± 0.13^{h}	4.8 ± 0.34^{h}	0.24 ± 0.02^{h}	9.6 ± 0.67^{h}	6.2 ± 0.43^{h}
GH3	124 ± 8.68^{e}	486 ± 34.02^{e}	386 ± 27.02^{e}	228 ± 15.96^{e}	92 ± 6.44^{e}	1.42 ± 0.11^{e}	5.0 ± 0.35^{e}	84 ± 5.88^{d}	17 ± 1.19^{f}	1.8 ± 0.14^{f}	5.4 ± 0.38^{f}	0.27 ± 0.02^{f}	12.0 ± 0.84^{f}	7.4 ± 0.52^{f}
GH4	149 ± 10.43^{c}	648 ± 45.36^{c}	418 ± 29.26^{d}	272 ± 19.04^{c}	118 ± 8.26^{c}	1.76 ± 0.14^{c}	6.2 ± 0.43^{c}	102 ± 7.14^{b}	18 ± 1.26^{de}	2.0 ± 0.16^{d}	6.0 ± 0.42^{cd}	0.30 ± 0.02^{cd}	14.8 ± 1.04^{d}	8.6 ± 0.60^{d}
GB	132 ± 9.24^{d}	592 ± 41.44^{d}	432 ± 30.24^{cd}	238 ± 16.66^{d}	108 ± 7.56^{d}	1.62 ± 0.13^{d}	5.6 ± 0.39^{d}	82 ± 5.74^{d}	17 ± 1.19^{f}	1.8 ± 0.14^{f}	5.1 ± 0.36^{fg}	0.27 ± 0.02^{f}	12.4 ± 0.87^{f}	7.0 ± 0.49^{f}
GR	118 ± 8.26^{e}	548 ± 38.36^{de}	398 ± 27.86^{de}	216 ± 15.12^{e}	96 ± 6.72^{e}	1.48 ± 0.12^{e}	4.8 ± 0.34^{e}	74 ± 5.18^{e}	16 ± 1.12^{g}	1.7 ± 0.14^{g}	4.8 ± 0.34^{h}	0.25 ± 0.02^{h}	11.6 ± 0.81^{g}	6.6 ± 0.46^{g}
GW	96 ± 6.72^{f}	412 ± 28.84^{f}	488 ± 34.16^{ab}	182 ± 12.74^{f}	74 ± 5.18^{f}	1.12 ± 0.09^{f}	4.2 ± 0.29^{f}	62 ± 4.34^{g}	19 ± 1.33^{cd}	1.9 ± 0.15^{e}	5.4 ± 0.38^{f}	0.30 ± 0.02^{cd}	13.8 ± 0.97^{e}	8.2 ± 0.57^{e}
GHB	166 ± 11.62^{b}	796 ± 55.72^{b}	452 ± 31.64^{bc}	314 ± 21.98^{b}	142 ± 9.94^{b}	2.06 ± 0.16^{b}	7.0 ± 0.49^{b}	110 ± 7.70^{ab}	20 ± 1.40^{bc}	2.1 ± 0.17^{c}	6.2 ± 0.43^{c}	0.33 ± 0.03^{bc}	16.4 ± 1.15^{b}	9.6 ± 0.67^{b}
PS	34 ± 2.38^{h}	106 ± 7.42^{h}	276 ± 19.32^{h}	68 ± 4.76^{h}	16 ± 1.12^{h}	0.40 ± 0.03^{h}	1.6 ± 0.13^{h}	22 ± 1.54^{i}	11 ± 0.77^{j}	1.1 ± 0.09^{j}	3.0 ± 0.24^{j}	0.17 ± 0.01^{j}	6.1 ± 0.43^{j}	4.0 ± 0.28^{j}
PH	88 ± 6.16^{g}	266 ± 18.62^{g}	344 ± 24.08^{g}	164 ± 11.48^{g}	56 ± 3.92^{g}	0.88 ± 0.07^{g}	3.2 ± 0.22^{g}	58 ± 4.06^{h}	14 ± 0.98^{i}	1.5 ± 0.12^{i}	4.4 ± 0.31^{i}	0.23 ± 0.02^{i}	8.9 ± 0.62^{i}	5.8 ± 0.41^{i}
PH2	106 ± 7.42^{f}	348 ± 24.36^{f}	366 ± 25.62^{f}	192 ± 13.44^{f}	70 ± 4.90^{f}	1.08 ± 0.09^{f}	4.0 ± 0.28^{f}	68 ± 4.76^{f}	15 ± 1.05^{h}	1.6 ± 0.13^{h}	4.9 ± 0.34^{h}	0.24 ± 0.02^{h}	10.0 ± 0.70^{h}	6.4 ± 0.45^{h}
PH3	129 ± 9.03^{de}	518 ± 36.26^{de}	392 ± 27.44^{de}	236 ± 16.52^{de}	96 ± 6.72^{e}	1.54 ± 0.12^{de}	5.2 ± 0.36^{e}	88 ± 6.16^{d}	18 ± 1.26^{de}	1.9 ± 0.15^{e}	5.6 ± 0.39^{e}	0.28 ± 0.02^{ef}	12.6 ± 0.88^{f}	7.6 ± 0.53^{f}

PH4	176 ± 12.32^{a}	852 ± 59.64^{a}	458 ± 32.06^{bc}	326 ± 22.82^{a}	152 ± 10.64^{a}	2.28 ± 0.18^{a}	7.6 ± 0.53^{a}	116 ± 8.12^{a}	22 ± 1.54^{a}	2.3 ± 0.18^{a}	7.0 ± 0.49^{a}	0.37 ± 0.03^{a}	18.2 ± 1.27^{a}	10.6 ± 0.74^{a}
PB	136 ± 9.52^{d}	624 ± 43.68^{cd}	426 ± 29.82^{cd}	246 ± 17.22^{d}	116 ± 8.12^{cd}	1.68 ± 0.13^{cd}	5.8 ± 0.41^{d}	86 ± 6.02^{d}	18 ± 1.26^{de}	1.8 ± 0.14^{f}	5.3 ± 0.37^{f}	0.28 ± 0.02^{ef}	12.8 ± 0.90^{f}	7.2 ± 0.50^{f}
PR	121 ± 8.47^{e}	574 ± 40.18^{d}	404 ± 28.28^{de}	224 ± 15.68^{e}	102 ± 7.14^{de}	1.56 ± 0.12^{de}	5.0 ± 0.35^{e}	76 ± 5.32^{e}	17 ± 1.19^{f}	1.7 ± 0.14^{g}	5.0 ± 0.35^{g}	0.26 ± 0.02^{g}	11.9 ± 0.83^{g}	6.8 ± 0.48^{g}
PW	102 ± 7.14^{f}	426 ± 29.82^{f}	502 ± 35.14^{ab}	188 ± 13.16^{f}	80 ± 5.60^{f}	1.18 ± 0.09^{f}	4.4 ± 0.31^{f}	66 ± 4.62^{f}	19 ± 1.33^{cd}	1.9 ± 0.15^{e}	5.7 ± 0.40^{e}	0.31 ± 0.02^{cd}	14.4 ± 1.01^{d}	8.6 ± 0.60^{d}
PRC	154 ± 10.78^{c}	706 ± 49.42^{c}	438 ± 30.66^{bc}	278 ± 19.46^{c}	128 ± 8.96^{c}	1.92 ± 0.15^{c}	6.4 ± 0.45^{c}	96 ± 6.72^{c}	20 ± 1.40^{bc}	2.0 ± 0.16^{d}	6.0 ± 0.42^{cd}	0.32 ± 0.03^{bc}	15.6 ± 1.09^{c}	9.0 ± 0.63^{c}
PFW	112 ± 7.84^{f}	462 ± 32.34^{ef}	524 ± 36.68^{a}	198 ± 13.86^{f}	86 ± 6.02^{f}	1.26 ± 0.10^{f}	4.6 ± 0.32^{f}	70 ± 4.90^{f}	21 ± 1.47^{ab}	2.2 ± 0.18^{b}	6.6 ± 0.46^{b}	0.35 ± 0.03^{b}	16.8 ± 1.18^{b}	10.0 ± 0.70^{b}
PHB	162 ± 11.34^{b}	744 ± 52.08^{b}	472 ± 33.04^{bc}	304 ± 21.28^{b}	138 ± 9.66^{b}	2.02 ± 0.16^{b}	6.8 ± 0.48^{b}	104 ± 7.28^{b}	19 ± 1.33^{bcd}	2.0 ± 0.16^{d}	6.1 ± 0.43^{c}	0.33 ± 0.03^{bc}	16.0 ± 1.12^{c}	9.4 ± 0.66^{c}
F-value	138.4	242.6	96.8	176.2	201.4	88.7	132.5	56.84	18.92	14.37	23.88	17.94	45.12	36.45
p-value	<0.001	<0.001	<0.001	<0.001	<0.001	<0.001	<0.001	<0.001	<0.001	<0.001	<0.001	<0.001	<0.001	<0.001
CV (%)	8.7	9.4	8.8	9.1	8.5	10.2	9	7.36	7.10	8.11	7.15	8.09	7.25	7.19
η^2	0.91	0.95	0.87	0.92	0.94	0.85	0.9	0.962	0.896	0.869	0.918	0.893	0.954	0.944
Range (min – max)	34–176	106–852	276–524	68–326	16–152	0.40–2.28	1.6–7.6	22–116	11–22	1.1–2.3	3.0–7.0	0.17–0.37	6.1–18.2	4.0–10.6
Overall mean	116.3	490	407.7	214.4	90.3	1.38	4.8	75.3	17.1	1.78	5.23	0.275	12.45	7.44

Values are expressed as mean ± standard deviation (SD) of three independent samples per experimental variant (n = 3). Each independent sample was measured using triplicate instrumental readings, which were averaged before statistical analysis. Different superscript lowercase letters within the same column indicate statistically significant differences among experimental variants according to one-way analysis of variance (ANOVA) followed by Duncan's multiple range test ($p < 0.05$). Samples sharing at least one identical superscript letter are not significantly different, whereas samples marked with different letters differ significantly at the 95% confidence level. One-way ANOVA revealed highly significant effects of the applied technological treatments on all investigated technological trace elements and potentially toxic elements ($p < 0.001$).

Table S4. Dietary exposure assessment based on estimated daily intake (EDI) and relative exposure increase calculated for the investigated experimental Romanian artisanal Pălincă-based beverages.

Sample	Technological category	EDI (mg/kg bw/day)	Relative exposure increase vs control (%)	Dominant exposure contributors	Exposure intensity classification
GS	Traditional grape Pălincă distillate	0.00084	Reference	Cu > Fe > Zn	Very low
GH	Honey-enriched grape Pălincă-based beverage	0.00112	+33.3	Cu > Fe > Zn > Mn	Low
GH2	Honey-enriched grape Pălincă-based beverage	0.00126	+50.0	Cu > Fe > Zn > Mn	Low
GH3	Honey-enriched grape Pălincă-based beverage	0.00149	+77.4	Fe > Cu > Zn > Mn	Moderate
GH4	Honey-enriched grape Pălincă-based beverage	0.00171	+103.6	Fe > Cu > Zn > Mn	Elevated
GB	Blackthorn-macerated grape Pălincă-based beverage	0.00158	+88.1	Cu > Zn > Fe	Elevated
GR	Rosehip-macerated grape Pălincă-based beverage	0.00144	+71.4	Fe > Cu > Zn	Moderate
GW	Wood-associated grape Pălincă-based beverage	0.00152	+81.0	Fe > Cu > Zn > Mn	Elevated
GHB	Mixed honey-botanical grape Pălincă-based beverage	0.00193	+129.8	Fe > Cu > Mn > Zn	High
PS	Traditional plum Pălincă distillate	0.00079	Reference	Cu > Fe > Zn	Very low
PH	Honey-enriched plum Pălincă-based beverage	0.00116	+46.8	Cu > Fe > Zn > Mn	Low
PH2	Honey-enriched plum Pălincă-based beverage	0.00131	+65.8	Fe > Cu > Zn > Mn	Moderate
PH3	Honey-enriched plum Pălincă-based beverage	0.00161	+103.8	Fe > Cu > Zn > Mn	Elevated
PH4	Honey-enriched plum Pălincă-based beverage	0.00208	+163.3	Fe > Cu > Mn > Zn	Highest
PHB	Mixed honey-botanical plum Pălincă-based beverage	0.00170	+115.2	Fe > Cu > Zn > Mn	Elevated
PB	Blackthorn-macerated plum Pălincă-based beverage	0.00167	+111.4	Cu > Zn > Fe	Elevated
PR	Rosehip-macerated plum Pălincă-based beverage	0.00151	+91.1	Fe > Cu > Zn	Moderate
PW	Wood-associated plum Pălincă-based beverage	0.00156	+97.5	Fe > Cu > Zn > Mn	Elevated

PRC	Intensified rosehip-botanical plum Pălincă-based beverage	0.00184	+132.9	Fe > Cu > Mn > Zn	High
PFW	Plum wood-associated botanical Pălincă-based beverage	0.00173	+119.0	Fe > Cu > Zn > Mn	Elevated

Note: EDI = estimated daily intake. Under the assumed daily exposure scenario, with EF = 365 days/year and AT = EF × ED, CDI was mathematically equivalent to EDI and was therefore not presented as a separate exposure metric to avoid redundancy. Relative exposure increase was calculated separately using GS as the control for grape-based variants and PS as the control for plum-based variants. Exposure intensity classes represent relative within-dataset classifications based on cumulative elemental intake and do not indicate toxicological risk categories.

Table S5. Integrated THQ- and HI-based non-carcinogenic elemental risk evaluation and comparative risk characterization of experimental Romanian artisanal Pălincă-based beverages.

**Sample**	**Technological category**	**Dominant toxicological contributors**	**Maximum THQ**	**Mean THQ**	**HI**	**Relative increase vs control (%)**	**Main associated technological factor**	**Overall elemental loading**	**Relative toxicological ranking**	Elemental risk interpretation
GS	Traditional grape Pălincă distillate	Cu > Fe > Zn	0.00533	0.00098	0.0137	Reference	Distillation equipment	Very low	Lowest	Low non-carcinogenic elemental risk
GH	Honey-enriched grape Pălincă-based beverage	Cu > Fe > Zn > Mn	0.00630	0.00137	0.0192	+40.1	Honey incorporation	Low	Very low	
GH2	Honey-enriched grape Pălincă-based beverage	Cu > Fe > Zn > Mn	0.00671	0.00156	0.0218	+59.1	Honey incorporation	Moderate	Low	
GH3	Honey-enriched grape Pălincă-based beverage	Fe > Cu > Zn > Mn	0.00724	0.00184	0.0258	+88.3	Honey incorporation	Moderate	Moderate	
GH4	Honey-enriched grape Pălincă-based beverage	Fe > Cu > Zn > Mn	0.00784	0.00212	0.0297	+116.8	Honey incorporation	Elevated	Elevated	
GB	Blackthorn-macerated grape Pălincă-based beverage	Cu > Zn > Fe	0.00810	0.00199	0.0279	+103.6	Fruit maceration	Elevated	Moderate	
GR	Rosehip-macerated grape Pălincă-	Fe > Cu > Zn	0.00746	0.00183	0.0256	+86.9	Rosehip extraction	Moderate	Moderate	

	based beverage									
GW	Wood-associated grape Pălincă-based beverage	Fe > Cu > Zn > Mn	0.00915	0.00189	0.0265	+93.4	Wood maturation	Elevated	Moderate	
GHB	Mixed honey-botanical grape Pălincă-based beverage	Fe > Cu > Mn > Zn	0.00848	0.00237	0.0333	+143.1	Honey + botanical matrices	High	High	Low non-carcinogenic elemental risk
PS	Traditional plum Pălincă distillate	Cu > Fe > Zn	0.00518	0.00092	0.0129	Reference	Distillation equipment	Very low	Lowest	
PH	Honey-enriched plum Pălincă-based beverage	Cu > Fe > Zn > Mn	0.00645	0.00141	0.0198	+53.5	Honey incorporation	Low	Very low	
PH2	Honey-enriched plum Pălincă-based beverage	Fe > Cu > Zn > Mn	0.00686	0.00159	0.0223	+72.9	Honey incorporation	Moderate	Low	
PH3	Honey-enriched plum Pălincă-based beverage	Fe > Cu > Zn > Mn	0.00735	0.00193	0.0270	+109.3	Honey incorporation	Elevated	Moderate	
PH4	Honey-enriched plum Pălincă-based beverage	Fe > Cu > Mn > Zn	0.00859	0.00254	0.0356	+175.9	Honey incorporation	Highest	Highest	
PHB	Mixed honey-botanical plum Pălincă-based beverage	Cu > Co > Mn > Pb	0.00885	0.00235	0.0328	+154.7	Honey + blackthorn fruits	High	High	

PB	Blackthorn-macerated plum Pălincă-based beverage	Cu > Zn > Fe	0.00799	0.00204	0.0285	+120.9	Blackthorn extraction	Elevated	Moderate
PR	Rosehip-macerated plum Pălincă-based beverage	Fe > Cu > Zn	0.00757	0.00189	0.0265	+105.4	Rosehip extraction	Moderate	Moderate
PW	Wood-associated plum Pălincă-based beverage	Fe > Cu > Zn > Mn	0.00941	0.00196	0.0274	+112.4	Wood maturation	Elevated	Moderate
PRC	Intensified rosehip-macerated plum Pălincă-based beverage	Fe > Cu > Mn > Zn	0.00821	0.00225	0.0315	+144.2	Intensified botanical extraction	High	High
PFW	Wood-associated combined-processing plum Pălincă-based beverage	Fe > Cu > Zn > Mn	0.00983	0.00212	0.0297	+130.2	Mulberry wood maturation	Elevated	Moderate

Notes: THQ = Target Hazard Quotient; Mean THQ = average individual elemental target hazard quotient calculated for all elements included in the HI model within each experimental sample; HI = Hazard Index, calculated as the sum of individual THQ values for the elements included in the risk model. In the present assessment, HI is equivalent to the total target hazard quotient (TTHQ), but HI is used as the standard term throughout the manuscript; Maximum individual THQ = highest individual elemental THQ contribution identified in each analyzed sample; Relative increase vs control (%) = percentage increase in cumulative HI values relative to the corresponding traditional control distillates (GS and PS). Dominant toxicological contributors represent the elements exhibiting the highest individual THQ contributions within each investigated sample. RfD values used for the risk assessment were as follows: Al = 1.000, Ba = 0.200, Cd = 0.0005, Co = 0.0003, Cr = 0.003, Cu = 0.040, Fe = 0.700, Li = 0.002, Mn = 0.024, Ni = 0.020, Se = 0.005, Sr = 0.600, Pb = 0.0035, and Zn = 0.300 mg/kg bw/day. Element-specific benchmark values were assigned according to the most relevant available oral non-carcinogenic reference values reported in EPA, WHO/FAO, ATSDR, and related regulatory toxicological guidance documents, with Pb treated as a conservative screening-level benchmark because no universally accepted safe exposure threshold is available for lead. Since ICP-MS provided total elemental concentrations, Cr was treated conservatively using a Cr(VI)-based RfD. Total As and total Hg were reported as analytical concentrations but were excluded from the cumulative HI calculation because

arsenic and mercury toxicity is strongly species-dependent and As/Hg speciation was not performed. Overall elemental loading and relative toxicological ranking represent comparative qualitative classifications based on cumulative HI intensity patterns observed among the investigated experimental Romanian artisanal Pălincă-based beverages. Honey incorporation, fruit maceration, wood-associated processing, and intensified botanical extraction procedures were identified as the principal technological factors associated with progressive increases in cumulative toxicological indices. Nevertheless, all calculated HI values remained substantially below the toxicological reference threshold of 1, indicating low or negligible chronic non-carcinogenic risk under the proposed exposure scenario. General note for Tables S6–S14: Numerical values reported in Tables S6–S14 were cross-checked against the primary datasets presented in Tables S1–S5. These tables provide interpretative syntheses only, and all reported ranges were harmonized with the corresponding raw analytical values.

Table S6. Integrated physicochemical, chromatic, functional, and elemental modifications induced by progressive honey enrichment in the experimental Romanian artisanal Pălincă-based beverages

Analytical category	Main observed results	Main implication
Alcoholic strength ↓	Honey-enriched variants showed lower final alcoholic strength, ranging from 46.8–47.1% v/v at the first enrichment level to 44.1–44.3% v/v in the highest honey-enriched variants.	Final matrix dilution associated with progressive honey incorporation
pH ↓	Reduced from 4.28–4.34 in controls to 3.89–3.93 in highly enriched variants	Increased matrix acidity and compositional complexity
Conductivity ↑	Increased from 186–194 μS/cm to 488–502 μS/cm	Higher ionic and mineral loading
Density / °Brix / Dry matter ↑	Density increased from 0.932–0.934 to 0.972–0.973 g/cm³; °Brix from 1.2–1.4 to 14.4–14.8; dry matter from 1.7–1.9 to 16.7–17.1 g/L	Transfer of sugars and dissolved organic compounds from honey
Turbidity ↑	Increased from 1.7–1.8 NTU in controls to 10.1–10.4 NTU in GH4 and PH4	Increased colloidal and suspended organic fraction
TPC / DPPH ↑	TPC increased from 38–42 to 304–338 mg GAE/L; DPPH from 16.2–18.4% to 62.8–68.2%	Enhanced antioxidant and functional profile
L* ↓ / b* ↑	L* decreased from 96.2–97.1 to 69.5–70.2, while b* increased from 2.1–2.4 to 25.7–26.4	Development of golden-yellow and amber coloration
K / Ca / Mg / Na ↑	K increased from 26.8–28.4 to 121.5–126.2 mg/L; Mg from 5.8–6.2 to 27.1–28.2 mg/L	Mineral enrichment associated with honey incorporation
Sr / Ba / Rb / Li / Cs ↑	Sr increased from 42–46 to 151–168 μg/L; Rb from 7.8–8.4 to 43.8–46.1 μg/L	More complex process-related elemental fingerprint
Fe / Cu / Zn / Mn ↑	Fe increased from 106–112 to 648–852 μg/L; Zn from 68–74 to 272–326 μg/L	Technological and botanical elemental enrichment
Pb / Cd / As / Hg / Ni / Cr ↑	Pb increased from 11–12 to 18–22 μg/L; Ni from 6.1–6.4 to 14.8–18.2 μg/L	Moderate elemental accumulation without critical toxicological risk1

Notes: ↑ indicates an increasing trend, whereas ↓ indicates a decreasing trend after progressive honey enrichment. GS and PS represent the control distillate variants, while GH, GH2, GH3, GH4 and PH, PH2, PH3, PH4 correspond to progressively honey-enriched experimental variants. TPC = total phenolic content; DPPH = radical scavenging activity; L* = lightness parameter; b* = yellow-blue chromatic coordinate according to the CIELAB color system. The presented data summarize the principal physicochemical, functional, chromatic, and elemental modifications observed after honey incorporation.

Table S7. Integrated overview of the principal chromatic, functional, physicochemical, and elemental modifications induced by fruit maceration in the experimental Romanian artisanal Pălincă-based beverages

Analytical category	Main observed results	Main implication
Chromatic profile (L*, a*, b*)	L* progressively decreased, while a* and b* values increased in the macerated variants compared to the control distillates	Development of darker amber, orange, and reddish chromatic tonalities associated with fruit pigment transfer
Turbidity ↑	Turbidity values increased in all macerated variants relative to the control samples	Increased suspended organic and colloidal fraction derived from fruit constituents
Density / °Brix / Dry matter ↑	Density, soluble solids, and dry matter content progressively increased after fruit maceration	Transfer of sugars, organic acids, and dissolved fruit compounds into the alcoholic matrix
TPC ↑	Total phenolic content increased markedly in the macerated variants compared to the controls	Enrichment with fruit-derived phenolic compounds and bioactive constituents
DPPH radical scavenging activity ↑	Antioxidant activity increased proportionally with maceration intensity	Improved antioxidant and functional potential of the beverages
Alcoholic strength ↓	Slight reduction of alcoholic strength observed after maceration	Dilution effect generated by fruit moisture and soluble fraction transfer
Conductivity ↑	Conductivity values progressively increased in the macerated variants	Increased ionic strength and mineral loading
K / Mg / Ca / Na ↑	Major mineral concentrations increased after fruit incorporation	Mineral enrichment associated with fruit-associated elemental transfer
Sr / Ba / Rb / Li / Cs ↑	Trace process-related elemental markers showed increased concentrations in the macerated variants	Development of a more complex process-related elemental fingerprint
Fe / Cu / Zn / Mn ↑	Technological and trace element concentrations increased moderately after maceration	Additional elemental contribution from fruits and technological processing

Notes: ↑ indicates an increasing trend, whereas ↓ indicates a decreasing trend after fruit maceration. TPC = total phenolic content; DPPH = radical scavenging activity; L* = lightness parameter; a* = red-green chromatic coordinate; b* = yellow-blue chromatic coordinate according to the CIELAB color system. The presented data summarize the principal compositional, chromatic, functional, and elemental modifications associated with fruit-derived compound transfer during the maceration process.

Table S8. Integrated overview of the principal physicochemical, oxidative, chromatic, functional, and elemental modifications induced by wood-assisted maturation in the experimental Romanian artisanal Pălincă-based beverages

Analytical category	Main observed results	Main implication
Chromatic profile (L*, a*, b*)	L* values progressively decreased, while a* and b* increased during maturation	Development of amber-brown coloration associated with oxidative aging and wood-derived pigment extraction
TPC ↑	Total phenolic content progressively increased in matured variants	Extraction of phenolic compounds, tannins, and lignin-derived constituents from wood
DPPH radical scavenging activity ↑	Antioxidant activity increased after maturation	Improved oxidative stability and functional profile
Conductivity ↑	Conductivity values increased during wood-assisted maturation	Increased ionic and mineral complexity
Density / Dry matter ↑	Density and dry matter content moderately increased after maturation	Accumulation of dissolved wood-derived organic compounds
Turbidity ↑	Slight increases in turbidity observed in matured samples	Formation of colloidal and polymerized oxidative structures
Alcoholic strength ↓	Moderate reduction in alcoholic strength after maturation	Volatilization and oxidative evaporation phenomena during aging
K / Ca / Mg / Na ↑	Major mineral concentrations increased after wood contact	Elemental enrichment associated with wood contact
Fe / Cu / Zn / Mn ↑	Trace technological elements progressively increased during maturation	Contribution of wood contact and oxidative technological processes
Sr / Ba / Rb / Li / Cs ↑	Process-related trace elements showed moderate increases	Development of a more complex maturation-associated elemental fingerprint
Oxidative transformations ↑	Progressive oxidative reactions occurred during maturation	Formation of more stable, complex, and mature compositional profile

Notes: ↑ indicates an increasing trend, whereas ↓ indicates a decreasing trend during wood-assisted maturation. TPC = total phenolic content; DPPH = radical scavenging activity; L* = lightness parameter; a* = red-green chromatic coordinate; b* = yellow-blue chromatic coordinate according to the CIELAB color system. The presented data summarize the principal physicochemical, oxidative, chromatic, functional, and elemental modifications associated with wood-assisted maturation processes in the investigated artisanal Pălincă-based beverages.

Table S9. Integrated overview of the principal ICP-MS elemental profiling patterns and technological discrimination features identified in the investigated Romanian artisanal Pălincă-based beverages

Analytical category	Main observed results	Main implication
Baseline elemental profile of control distillates	Control variants produced in the Tinca area (GS and PS) exhibited relatively stable baseline concentrations of Sr (42–46 µg/L), Rb (7.8–8.4 µg/L), Li (0.79–0.84 µg/L), and Cs (0.08–0.09 µg/L)	Baseline compositional reference for comparison with enriched and processed variants
Raw material differentiation	GS exhibited higher K (28.4 mg/L), Mg (6.2 mg/L), and Rb (8.4 µg/L) compared with PS, confirming elemental differences between grape- and plum-based distillates	Differentiation according to fruit substrate and botanical origin
Honey enrichment markers	In GH4 and PH4, K increased to 121.5–126.2 mg/L, Mg to 27.1–28.2 mg/L, Na reached 22.4 mg/L in GH4 and 25.0 mg/L in PH4, and Rb to 43.8–46.1 µg/L	Identification of honey incorporation and strong mineral enrichment
Progressive chromatic-associated elemental enrichment	Progressive chromatic-associated elemental enrichment, amber/deep golden variants showed increased Fe and Zn, with maximum values in GH4 and PH4: Fe 648–852 µg/L and Zn 272–326 µg/L, link between oxidative coloration and elemental enrichment	Association between oxidative coloration and elemental enrichment
Fruit maceration fingerprint	Blackthorn- and rosehip-macerated variants (GB, PB, GR, PR, PRC) showed increased Fe, Zn, Mn, Ba, and Sr concentrations relative to GS and PS	Elemental enrichment associated with fruit maceration and increased elemental complexity
Intensified botanical maceration effect	PRC exhibited higher elemental loading compared with standard PR due to intensified fragmented rosehip maceration	Enhanced extraction efficiency associated with intensified botanical processing
Wood maturation markers	GW, PW, and PFW variants exhibited progressive increases in K, Ca, Fe, Cu, and Mn concentrations after wood-assisted maturation	Development of maturation-associated elemental signature and oxidative evolution
Mulberry wood maturation influence	PFW exhibited darker coloration and higher overall mineral complexity compared with PW	Influence of wood type on elemental migration and maturation profile
Technological contamination markers	Fe concentrations reached the highest values in the strongly honey-enriched variants GH4 and PH4, ranging from 648 to 852 µg/L, while wood-associated variants showed moderate Fe enrichment.	Influence of traditional copper distillation systems and technological processing
Trace process-related elements (Sr, Rb, Li, Cs)	Sr increased from 42–46 to 151–168 µg/L, while Rb increased from 7.8–8.4 to 43.8–46.1 µg/L in highly enriched variants	Potential applicability for traceability-oriented interpretation and chemometric classification

Mixed honey–botanical Pălincă-based beverage profile	GHB and PHB exhibited the highest overall elemental diversity due to simultaneous honey enrichment and fruit maceration	Increased compositional complexity associated with combined technological procedures

Notes: ↑ indicates an increasing trend in elemental complexity. ICP-MS = inductively coupled plasma mass spectrometry. GS and PS represent the control distillates; GH–GH4 and PH–PH4 represent honey-enriched variants; GB, GR, PB, and PR represent simple fruit-macerated variants; PRC represents an intensified rosehip-macerated plum variant assigned to the mixed/intensified botanical category; GW and PW represent wood-only associated variants; PFW represents a wood-associated combined-processing variant; and GHB and PHB represent mixed honey-botanical variants. Therefore, PRC, PFW, GHB, and PHB should be interpreted as combined or intensified processing variants rather than as simple single-treatment samples. The presented data summarize the principal elemental profiling patterns, ingredient-related differentiation features, and technological discrimination characteristics identified among the investigated experimental artisanal Pălincă-based beverages produced in the Tinca area, Bihor County, north-western Romania.

Table S10. Integrated overview of the principal technological trace elements, contamination pathways, and processing-associated elemental modifications identified in the investigated Romanian artisanal Pălincă-based beverages

Analytical category	Main observed results	Main implication
Baseline technological elemental profile	Control variants (GS and PS) exhibited the lowest Fe (106–112 µg/L) and comparatively reduced Cu concentrations	Limited technological contamination under traditional double-distillation conditions
Fe technological enrichment	Fe concentrations reached the highest values in the strongly honey-enriched variants GH4 and PH4, ranging from 648 to 852 µg/L, while GH3, PH3, GW, PW, and PFW showed moderate Fe enrichment relative to the control distillates	Influence of technological processing, oxidative evolution, and metallic contact surfaces
Cu distillation markers	Cu concentrations progressively increased in technologically processed variants	Possible Cu contribution from traditional copper distillation systems or contact surfaces
Zn and Mn accumulation	Enriched and macerated variants exhibited higher Zn and Mn concentrations compared with controls	Combined influence of botanical enrichment and technological processing
Fruit maceration-associated elemental transfer	GB, PB, GR, PR, and PRC showed increased Fe, Zn, Mn, Ba, and Sr concentrations	Mineral transfer from fruit tissues during prolonged maceration
Intensified maceration effect	PRC exhibited higher overall elemental loading than standard PR	Increased extraction efficiency after fragmented rosehip maceration
Wood maturation-associated elemental migration	GW, PW, and PFW exhibited progressive increases in Fe, Cu, Mn, K, and Ca after maturation	Influence of oxidative aging and wood/contact-associated mineral transfer
Mulberry wood maturation influence	PFW showed darker coloration and greater mineral complexity compared with PW	Influence of wood type on maturation-associated elemental profile
Pb and Ni technological variability	Pb ranged between 11–22 µg/L, while Ni varied between 6.1–18.2 µg/L across the investigated variants	Moderate technological and environmental elemental contribution without critical toxicological risk
Cr, Cd, and As occurrence	Cr, Cd, and As exhibited moderate increases in technologically processed variants	Potential cumulative contribution from raw materials, equipment, and processing conditions

Notes: GS and PS represent the traditional control distillates. GH–GH4 and PH–PH4 represent honey-enriched variants; GB, GR, PB, and PR represent simple fruit-macerated variants; PRC represents an intensified rosehip-macerated plum variant assigned to the mixed/intensified botanical category; GW and PW represent wood-only associated variants; PFW represents a wood-associated combined-processing variant and should not be interpreted as a wood-only effect; and GHB and PHB represent mixed honey-botanical variants. ICP-MS = inductively coupled plasma mass spectrometry. The presented data summarize the principal technological trace element patterns, contamination-associated pathways, and processing-related elemental modifications identified among the investigated Romanian artisanal Pălincă-based beverages produced in the Tinca area (Bihor County, north-western Romania).

Table S11. Integrated overview of the principal potentially toxic elements, technological accumulation patterns, and elemental safety and non-carcinogenic risk assessment parameters identified in the investigated Romanian artisanal Pălincă-based beverages

Analytical category	Main observed results	Main implication
Pb occurrence	Pb concentrations ranged between 11–22 μg/L across all investigated variants	Moderate Pb occurrence without critical toxicological risk
Cd occurrence	Cd concentrations remained within low concentration ranges in all samples	Limited cadmium accumulation associated with technological processing
As occurrence	As concentrations exhibited moderate increases in enriched and matured variants	Possible contribution from raw materials and environmental background
Hg occurrence	Hg concentrations remained at very low levels in all investigated variants	Negligible mercury-associated toxicological concern
Ni and Cr variability	Ni ranged between 6.1–18.2 μg/L, while Cr exhibited moderate technological variability	Influence of processing equipment and technological contact surfaces
Technological accumulation trend	Enriched, matured, and mixed variants exhibited higher elemental loading than controls	Progressive accumulation associated with technological complexity
Honey enrichment toxicological effect	GH and PH variants exhibited moderate increases in Pb, Ni, and As relative to controls	Elemental enrichment associated with honey incorporation
Fruit maceration-associated toxicological markers	GB, PB, GR, PR, and PRC exhibited increased Fe, Zn, Mn, and moderate increases in Pb and Ni	Additional elemental extraction during prolonged maceration
Wood maturation-associated elemental enrichment	GW, PW, and PFW exhibited increased Fe, Cu, Ni, and Cr concentrations after maturation	Elemental changes associated with wood contact and oxidative processing
Mixed honey-botanical variant complexity	GHB and PHB exhibited the highest cumulative elemental loading among all investigated variants	Combined influence of multiple technological processes
THQ and HI evaluation	All THQ and HI values remained below the critical threshold value of 1	Absence of significant non-carcinogenic human health risk
Overall non-carcinogenic elemental risk interpretation	All investigated variants showed low non-carcinogenic elemental risk under the proposed moderate-consumption scenario	Preservation of an acceptable non-carcinogenic elemental safety profile despite technological enrichment processes

Notes: THQ = target hazard quotient; HI = Hazard Index, calculated as the sum of individual THQ values. GS and PS represent the traditional control distillates, while GH/PH, GB/PB, GR/PR/PRC, GW/PW/PFW, and GHB/PHB correspond to honey-enriched, fruit-macerated, intensified botanical maceration, wood-associated, and mixed Pălincă-based beverages, respectively. The presented data summarize the principal toxicological characteristics, technological accumulation trends, and elemental exposure and non-carcinogenic risk assessment parameters identified among the investigated Romanian artisanal Pălincă-based beverages.

Table S12. Integrated overview of the principal non-carcinogenic human health risk assessment parameters, exposure characteristics, and toxicological safety indicators identified in the investigated Romanian artisanal Pălincă-based beverages based on THQ and HI evaluation models

Analytical category	Main observed results	Main implication
Control variant exposure profile	GS and PS exhibited the lowest EDI, THQ, and HI values among all investigated variants	Minimal non-carcinogenic exposure risk under moderate consumption conditions
Honey enrichment exposure trend	GH and PH variants exhibited progressive increases in EDI, THQ, and HI values with increasing honey incorporation	Incremental elemental exposure associated with mineral enrichment processes
Fruit maceration-associated exposure	GB, PB, GR, PR, and PRC variants exhibited moderate increases in cumulative elemental exposure	Additional trace element transfer during prolonged fruit maceration
Intensified maceration toxicological effect	PRC exhibited higher cumulative exposure indices compared with standard PR	Increased extraction efficiency associated with intensified botanical processing
Wood maturation-associated exposure	GW, PW, and PFW variants exhibited moderate increases in THQ and HI values after maturation	Contribution of oxidative maturation and wood-derived elemental migration
Mixed honey-botanical variant exposure complexity	GHB and PHB exhibited high cumulative elemental exposure and ranked among the most enriched variants; however, PH4 showed the highest EDI and HI values in the dataset	Combined influence of enrichment, maceration, and maturation procedures
Maximum THQ values	Maximum THQ values remained substantially below the critical threshold value of 1 in all variants	Absence of significant individual elemental non-carcinogenic risk
HI evaluation	All HI values remained below the safety threshold value of 1	Low modeled cumulative non-carcinogenic elemental risk under the defined exposure scenario
Dominant toxicological contributors	Fe, Cu, Zn, Mn, Pb, and Ni represented the principal contributors to cumulative exposure indices	Major influence of technological and enrichment-associated elements
Relative toxicological ranking	Control variants exhibited the lowest toxicological ranking, while mixed and highly enriched variants exhibited the highest	Progressive increase in exposure associated with technological complexity
Overall non-carcinogenic elemental risk interpretation	All investigated artisanal Pălincă-based beverages showed low non-carcinogenic elemental risk under the proposed moderate-consumption scenario	Low non-carcinogenic elemental risk profile under the modeled exposure scenario

Notes: EDI = estimated daily intake; THQ = target hazard quotient; HI = hazard index. GS and PS represent the traditional control distillates, while GH/PH, GB/PB, GR/PR/PRC, GW/PW/PFW, and GHB/PHB correspond to honey-enriched, fruit-macerated, intensified botanical maceration, wood-associated, and mixed Pălincă-based beverages, respectively. The presented data summarize the principal non-carcinogenic exposure characteristics, toxicological safety indicators, and cumulative human health risk assessment parameters identified among the investigated Romanian artisanal Pălincă-based beverages.

Table S13. Integrated overview of the principal correlation patterns and chemometric differentiation features identified among the experimental Romanian artisanal Pălincă-based beverages

Analytical category	Main observed results	Main implication
Physicochemical–functional correlations	Conductivity, °Brix, dry matter, turbidity, TPC, and DPPH showed positive associations in enriched and macerated variants	Common contribution of dissolved organic matter, minerals, phenolics, and antioxidant compounds
Alcoholic strength correlation	Alcoholic strength was negatively associated with °Brix, dry matter, density, conductivity, TPC, and DPPH	Dilution and enrichment effect generated by honey incorporation, fruit maceration, and maturation
Chromatic correlations	L* decreased as a*, b*, TPC, turbidity, and elemental loading increased	Development of darker amber, reddish, orange, and brown tonalities associated with pigment transfer and oxidative evolution
Honey-enriched variant clustering	GH and PH variants grouped separately from GS and PS due to increased K, Mg, Na, Rb, TPC, °Brix, and dry matter	Clear chemometric discrimination of honey incorporation
Fruit-macerated variant clustering	GB, PB, GR, PR, and PRC were differentiated by increased chromatic intensity, TPC, DPPH, Fe, Zn, Mn, Ba, and Sr	Identification of fruit-derived compositional and elemental fingerprints
wood-associated variant clustering	GW, PW, and PFW were separated by higher oxidative markers, darker coloration, Fe, Cu, Mn, K, and Ca	Discrimination of maturation-associated technological fingerprints
Mixed honey-botanical variant differentiation	GHB and PHB showed the highest compositional complexity and occupied distinct chemometric positions	Combined influence of honey enrichment, fruit maceration, and technological processing
Process-related elemental correlations	Sr, Rb, Li, Cs, K, Mg, and Ba contributed to sample differentiation according to technological category	Applicability of trace elements as preliminary authenticity-related and classification markers

Notes: PCA = principal component analysis; TPC = total phenolic content; DPPH = radical scavenging activity; L* = lightness parameter; a* = red-green chromatic coordinate; b* = yellow-blue chromatic coordinate according to the CIELAB color system. GS and PS represent the traditional control distillates, while GH/PH, GB/PB, GR/PR/PRC, GW/PW/PFW, and GHB/PHB correspond to honey-enriched, fruit-macerated, intensified botanical maceration, wood-associated, and mixed Pălincă-based beverages, respectively. The presented data summarize the principal correlation patterns and chemometric differentiation features observed among the investigated Romanian artisanal Pălincă-based beverages.

Table S14. Integrated overview of the principal technological significance, preliminary authenticity-related potential, and functional valorization characteristics identified in the newly developed Romanian artisanal Pălincă-based beverages

Analytical category	Main observed results	Main implication
Honey enrichment functional effect	GH and PH variants exhibited increased TPC, DPPH, conductivity, °Brix, dry matter, K, Mg, and Rb concentrations	Development of functional beverages with enhanced antioxidant and mineral profile
Fruit maceration valorization potential	GB, PB, GR, PR, and PRC variants showed intensified chromatic profile, increased phenolic content, and higher elemental complexity	Valorization of spontaneous local botanical resources through artisanal beverage diversification
Wood maturation technological significance	GW, PW, and PFW exhibited darker coloration, oxidative evolution, and increased Fe, Cu, Mn, K, and Ca concentrations	Development of maturation-associated sensory and compositional complexity
Intensified botanical extraction effect	PRC exhibited higher phenolic and elemental loading compared with standard PR	Increased extraction efficiency and enhanced functional profile
Mixed honey-botanical beverage complexity	GHB and PHB exhibited the highest overall compositional diversity among all investigated variants	Synergistic effect of combined enrichment, maceration, and technological processing
Antioxidant functional enhancement	TPC increased from 38–42 mg GAE/L in controls to 304–338 mg GAE/L in highly enriched variants, while DPPH increased from 16.2–18.4% to 62.8–68.2%	Substantial improvement of antioxidant potential and bioactive profile
Traceability-oriented elemental fingerprint	Sr, Rb, Li, Cs, K, Mg, and Ba exhibited clear technological and compositional differentiation patterns	Potential applicability for preliminary authenticity-related and traceability studies
Regional raw material valorization	All variants were produced using raw materials originating from the Tinca area (Bihor County, Romania)	Preservation of territorial identity and local artisanal specificity
Traditional technology preservation	Experimental beverages maintained traditional double-distillation and artisanal processing procedures	Conservation of traditional Romanian distillation practices
Elemental safety preservation	All THQ and HI values remained below the critical threshold value of 1	Preservation of an acceptable non-carcinogenic elemental safety profile despite technological diversification
ICP-MS technological applicability	Elemental profiling clearly differentiated enriched, macerated, matured, and mixed variants	Applicability of ICP-MS for technological characterization and preliminary authenticity-supporting interpretation
Functional and commercial valorization potential	Newly developed variants combined traditional artisanal specificity with	Potential development of premium-value artisanal functional distilled beverages

improved antioxidant and compositional characteristics

Notes: TPC = total phenolic content; DPPH = radical scavenging activity; THQ = target hazard quotient; HI = hazard index/total target hazard quotient; ICP-MS = inductively coupled plasma mass spectrometry. GS and PS represent the traditional control distillates, while GH/PH, GB/PB, GR/PR/PRC, GW/PW/PFW, and GHB/PHB correspond to honey-enriched, fruit-macerated, intensified botanical maceration, wood-associated, and mixed Pălincă-based beverages, respectively. The presented data summarize the principal technological, functional, authenticity-related, and valorization characteristics identified among the investigated experimental Romanian artisanal Pălincă-based beverages.

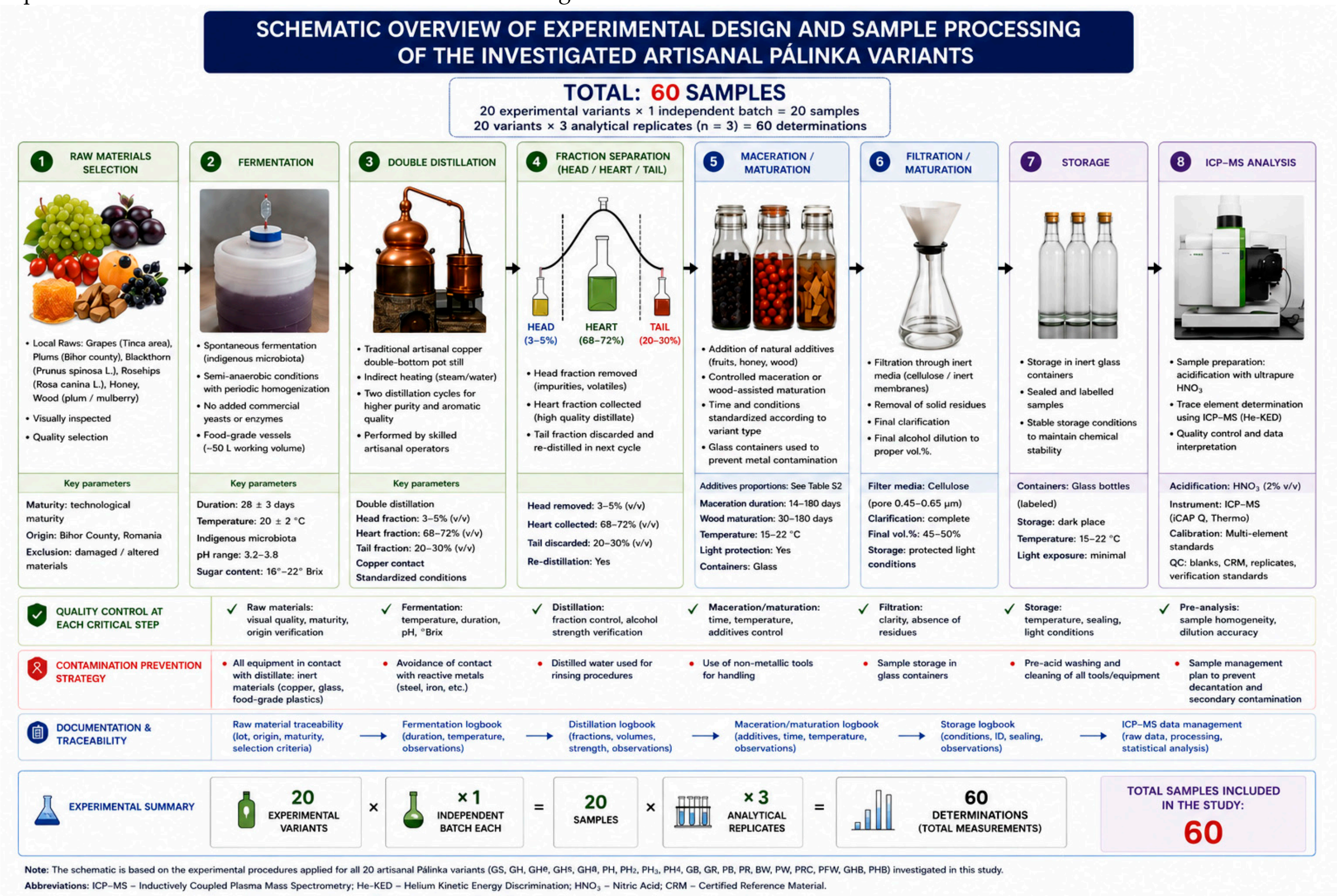

Figure S1. Supplementary process map of the experimental design, sample processing, quality-control checkpoints, contamination-prevention strategy, traceability records, and analytical allocation used for the investigated Romanian artisanal Pălincă-based beverages.

Table S15. Experimental classification, sample coding, and technological categories of the investigated artisanal Pălincă-based beverages.

Sample Code	Experimental Variant	Base Raw Material	Natural Additive / Maturation Material	Experimental Category
GS	Traditional grape Pălincă distillate	Grapes (*Vitis vinifera* L.)	None	Traditional Pălincă distillate
GH	Experimental grape Pălincă-based beverage with honey		Artisanal polyfloral honey (1/3, v/v)	Honey-enriched Pălincă-based beverage
GH2	Experimental grape Pălincă-based beverage with honey		Artisanal polyfloral honey (1/2, v/v)	Honey-enriched Pălincă-based beverage
GH3	Experimental grape Pălincă-based beverage with honey		Artisanal polyfloral honey (2/3, v/v)	Honey-enriched Pălincă-based beverage
GH4	Experimental grape Pălincă-based beverage with honey		Artisanal polyfloral honey (3/4, v/v)	Honey-enriched Pălincă-based beverage
GB	Experimental grape Pălincă-based beverage macerated with blackthorn fruits		Blackthorn fruits (*Prunus spinosa* L.)	Fruit-macerated Pălincă-based beverage
GR	Experimental grape Pălincă-based beverage macerated with rosehips		Rosehips (*Rosa canina* L.)	Fruit-macerated Pălincă-based beverage
GW	Experimental grape Pălincă-based beverage matured with plum wood		Plum wood fragments	Wood-associated Pălincă-based beverage
GHB	Experimental grape Pălincă-based beverage with honey and blackthorn fruits		Honey + blackthorn fruits	Mixed honey–botanical Pălincă-based beverage
PS	Traditional plum Pălincă distillate	Plums (*Prunus domestica* L.)	None	Traditional Pălincă distillate
PH	Experimental plum Pălincă-based beverage with honey		Artisanal polyfloral honey (1/3, v/v)	Honey-enriched Pălincă-based beverage
PH2	Experimental plum Pălincă-based beverage with honey		Artisanal polyfloral honey (1/2, v/v)	Honey-enriched Pălincă-based beverage

PH3	Experimental plum Pălincă-based beverage with honey	Artisanal polyfloral honey (2/3, v/v)	Honey-enriched Pălincă-based beverage
PH4	Experimental plum Pălincă-based beverage with honey	Artisanal polyfloral honey (3/4, v/v)	Honey-enriched Pălincă-based beverage
PB	Experimental plum Pălincă-based beverage macerated with blackthorn fruits	Blackthorn fruits (*Prunus spinosa* L.)	Fruit-macerated Pălincă-based beverage
PR	Experimental plum Pălincă-based beverage macerated with rosehips	Rosehips (*Rosa canina* L.)	Fruit-macerated Pălincă-based beverage
PW	Experimental plum Pălincă-based beverage matured with plum wood	Plum wood fragments	Wood-associated Pălincă-based beverage
PRC	Experimental plum Pălincă-based beverage with fragmented rosehips	Fragmented rosehip fruits	Intensified botanical Pălincă-based beverage
PFW	Experimental plum Pălincă-based beverage with rosehips and mulberry wood fragments	Rosehips + mulberry wood fragments	Wood-associated combined-processing Pălincă-based beverage
PHB	Experimental plum Pălincă-based beverage with honey and blackthorn fruits	Honey + blackthorn fruits	Mixed honey–botanical Pălincă-based beverage

Table S16. Standardized additive proportions and maceration/maturation periods applied for the preparation of the experimental artisanal Pălincă-based beverages produced in the Tinca area, Bihor County, north-western Romania.

Sample Code	Additive / Maturation Material	Additive Proportion	Initial Alcoholic Strength before Additive Incorporation (% v/v)	Maceration / Maturation Period
GS	None	—	Original; not adjusted	—
GH	Artisanal polyfloral honey	1/3 (v/v)	≈50	21 days
GH2	Artisanal polyfloral honey	1/2 (v/v)	≈50	21 days
GH3	Artisanal polyfloral honey	2/3 (v/v)	≈50	21 days
GH4	Artisanal polyfloral honey	3/4 (v/v)	≈50	21 days
GB	Blackthorn fruits (*Prunus spinosa* L.)	100 g/L	≈50	30 days
GR	Rosehips (*Rosa canina* L.)	100 g/L	≈50	30 days
GW	Plum wood fragments	20 g/L	≈50	45 days
GHB	Honey + blackthorn fruits	Honey: 1/3 (v/v) + 70 g/L blackthorn	≈50	30 days
PS	None	—	Original; not adjusted	—
PH	Artisanal polyfloral honey	1/3 (v/v)	≈50	21 days
PH2	Artisanal polyfloral honey	1/2 (v/v)	≈50	21 days
PH3	Artisanal polyfloral honey	2/3 (v/v)	≈50	21 days
PH4	Artisanal polyfloral honey	3/4 (v/v)	≈50	21 days
PB	Blackthorn fruits (*Prunus spinosa* L.)	100 g/L	≈50	30 days
PR	Rosehips (*Rosa canina* L.)	100 g/L	≈50	30 days
PW	Plum wood fragments	20 g/L	≈50	45 days

PRC	Fragmented rosehips (*Rosa canina* L.)	150 g/L	≈50	45 days
PFW	Rosehips + mulberry wood fragments	120 g/L rosehips + 20 g/L mulberry wood fragments	≈50	45 days
PHB	Honey + blackthorn fruits	Honey: 1/3 (v/v) + 70 g/L blackthorn	≈50	30 days

Notes: Distillate aliquots used for the preparation of the experimental Pălincă-based beverages obtained by additive incorporation or maturation-associated processing were adjusted to a target alcoholic strength of approximately 50% v/v before additive incorporation. The term "approximately" was used because this adjustment represented a practical pre-processing standardization step under artisanal conditions, where minor deviations may result from volumetric adjustment, temperature-dependent alcohol measurement, and rounding of alcoholic strength values. After additive incorporation and maceration/maturation, the final alcoholic strength of the variants could further change depending on honey addition, fruit maceration, or wood-associated processing. The untreated control distillates GS and PS were analyzed at their original alcoholic strengths and were not adjusted prior to analysis.

Table S17. Pre-treatment procedures and fragmentation characteristics of the botanical and maturation materials used for the preparation of the experimental artisanal Pălincă-based beverages.

Material	Pre-Treatment	Fragment Size
Blackthorn fruits (*Prunus spinosa* L.)	Visually inspected, washed with distilled water, air-dried prior to maceration	Whole fruits and fragmented material (approximately 1–2 cm)
Rosehips (*Rosa canina* L.)	Washed with distilled water, manually cleaned, air-dried prior to processing	
Fragmented rosehips (*Rosa canina* L.)	Distilled water washing, manual fragmentation, controlled air-drying	Standardized fragmented particles (~0.5–1.5 cm)
Plum wood fragments	Mechanical cleaning, air-drying, removal of degraded external surfaces	Standardized wood fragments (~2–5 cm length)
Mulberry wood fragments	Mechanical surface cleaning and controlled drying	
Artisanal polyfloral honey	Filtration through inert food-grade filtration material	Homogeneous liquid phase

Table S18. Comprehensive ICP-MS analytical validation, calibration performance, sensitivity parameters, precision, uncertainty estimation, and quality assurance criteria applied for the multi-elemental characterization of the investigated Romanian artisanal Pălincă-based beverages

Element	Isotope monitored	Analytical mode	Calibration range	Calibration points	R^2	Slope	LoD	LoQ	Recovery (%)	Intra-day RSD (%)	Inter-day RSD (%)	Expanded uncertainty (%)	Instrument drift (%)	Background equivalent concentration	CRM agreement (%)	Internal standard	Collision mode
Li	^{7}Li	Standard	0.05–100 µg/L	6	0.9997	0.998	0.004 µg/L	0.013 µg/L	96.4	3.1	4.2	5.3	1.8	0.002 µg/L	97.2	Sc	He-KED
Na	^{23}Na	Standard	0.005–20 mg/L	6	0.9992	0.994	0.008 mg/L	0.027 mg/L	94.8	4.6	5.7	6.8	2.5	0.005 mg/L	95.4	Sc	He-KED
Mg	^{24}Mg	Standard	0.005–20 mg/L	6	0.9995	0.997	0.004 mg/L	0.014 mg/L	97.2	3.8	4.9	5.9	2.1	0.003 mg/L	98.1	Sc	He-KED
Al	^{27}Al	Collision cell	0.1–1000 µg/L	7	0.9991	0.993	0.061 µg/L	0.203 µg/L	93.4	6.8	8.1	9.6	3.8	0.021 µg/L	94.2	Ge	He-KED
K	^{39}K	Standard	0.01–50 mg/L	7	0.9988	0.991	0.018 mg/L	0.060 mg/L	93.6	5.2	6.4	7.7	3.2	0.009 mg/L	94.3	Sc	He-KED
Ca	^{44}Ca	Standard	0.01–50 mg/L	7	0.9991	0.993	0.021 mg/L	0.070 mg/L	95.1	4.9	6.0	7.1	2.8	0.011 mg/L	95.8	Sc	He-KED

Cr	^{52}Cr	Collision cell	0.05–100 μg/L	6	0.9994	0.996	0.018 μg/L	0.060 μg/L	91.7	6.4	7.5	8.9	3.4	0.007 μg/L	92.6	Ge	He-KED
Mn	^{55}Mn	Standard	0.05–250 μg/L	6	0.9998	0.999	0.006 μg/L	0.020 μg/L	98.3	2.7	3.5	4.4	1.5	0.003 μg/L	99.0	Ge	He-KED
Fe	^{56}Fe	Collision cell	0.1–1000 μg/L	7	0.9990	0.992	0.074 μg/L	0.247 μg/L	92.8	6.9	8.3	9.5	3.9	0.025 μg/L	93.5	Ge	He-KED
Co	^{59}Co	Collision cell	0.005–50 μg/L	6	0.9997	0.998	0.003 μg/L	0.010 μg/L	97.3	3.7	4.6	5.4	2.0	0.001 μg/L	98.1	Ge	He-KED
Ni	^{60}Ni	Collision cell	0.02–100 μg/L	6	0.9996	0.998	0.011 μg/L	0.037 μg/L	96.9	4.1	5.2	6.1	2.3	0.004 μg/L	97.4	Ge	He-KED
Cu	^{63}Cu	Standard	0.05–500 μg/L	6	0.9997	0.998	0.009 μg/L	0.030 μg/L	97.6	3.5	4.6	5.5	2.0	0.003 μg/L	98.2	Rh	He-KED
Zn	^{66}Zn	Collision cell	0.1–1000 μg/L	7	0.9993	0.995	0.052 μg/L	0.173 μg/L	94.2	7.3	8.7	10.2	4.1	0.018 μg/L	95.0	Rh	He-KED
As	^{75}As	Collision cell	0.01–50 μg/L	6	0.9995	0.997	0.007 μg/L	0.023 μg/L	95.8	4.8	5.9	7.0	2.7	0.003 μg/L	96.3	Ge	He-KED
Se	^{78}Se	Collision cell	0.01–100 μg/L	6	0.9993	0.995	0.012 μg/L	0.040 μg/L	94.6	5.9	7.1	8.3	3.1	0.005 μg/L	95.2	Ge	He-KED
Rb	^{85}Rb	Standard	0.01–100 μg/L	6	0.9998	0.999	0.003 μg/L	0.010 μg/L	99.1	2.4	3.1	4.0	1.2	0.001 μg/L	99.4	Rh	He-KED

Sr	^{88}Sr	Standard	0.01–250 µg/L	6	0.9999	1.000	0.002 µg/L	0.007 µg/L	98.7	2.1	2.9	3.6	1.1	0.001 µg/L	99.1	Rh	He-KED
Cd	^{111}Cd	Collision cell	0.005–25 µg/L	6	0.9998	0.999	0.002 µg/L	0.006 µg/L	97.9	3.6	4.4	5.1	1.9	0.001 µg/L	98.5	In	He-KED
Cs	^{133}Cs	Standard	0.005–50 µg/L	6	0.9999	1.000	0.001 µg/L	0.004 µg/L	99.3	2.0	2.8	3.5	1.0	0.0004 µg/L	99.6	In	He-KED
Ba	^{138}Ba	Standard	0.01–250 µg/L	6	0.9996	0.998	0.010 µg/L	0.033 µg/L	96.1	4.5	5.4	6.6	2.4	0.004 µg/L	97.0	In	He-KED
Hg	^{202}Hg	Collision cell	0.005–20 µg/L	6	0.9991	0.992	0.004 µg/L	0.013 µg/L	89.4	8.6	9.8	11.5	4.8	0.002 µg/L	90.7	In	He-KED
Pb	^{208}Pb	Collision cell	0.005–50 µg/L	6	0.9997	0.999	0.003 µg/L	0.010 µg/L	97.1	3.9	4.8	5.7	2.2	0.001 µg/L	98.0	Bi/In	He-KED

Notes: R^2 = coefficient of determination of the calibration curve; LoD = limit of detection; LoQ = limit of quantification; RSD = relative standard deviation; CRM = certified reference material; He-KED = helium kinetic energy discrimination mode; BEC = background equivalent concentration. Calibration curves were generated using multi-element external calibration standards prepared under identical analytical conditions using acidified ultrapure solutions containing approximately 2% HNO_3 (v/v). Multi-point calibration models consisting of six or seven concentration levels were applied depending on the elemental concentration interval and analytical sensitivity requirements of each investigated analyte. Calibration linearity was considered acceptable when the correlation coefficient exceeded $R^2 \geq 0.995$. Limits of detection (LoD) and limits of quantification (LoQ) were calculated as three times and ten times the standard deviation of procedural blank measurements (3σ and 10σ criteria), respectively. Background equivalent concentration (BEC) values were estimated in order to evaluate instrumental background contribution and analytical sensitivity for each monitored isotope. Recovery percentages were determined through spike-recovery experiments using fortified analytical solutions and matrix-matched samples prepared at different concentration levels. Recovery values ranging between 85–115% were considered analytically acceptable according to internationally applied ICP-MS quality assurance criteria for food and beverage matrices. Intra-day precision was evaluated through repeated triplicate analyses performed within the same analytical sequence, whereas inter-day precision was assessed during three consecutive analytical days under identical operating conditions. Precision performance was expressed as relative standard deviation (%RSD), and values below 10% were considered indicative of satisfactory analytical repeatability and reproducibility. Expanded measurement uncertainty was estimated at a 95% confidence interval considering cumulative contributions from calibration variability, procedural repeatability, instrumental drift, recovery efficiency, and sample preparation uncertainty. Instrumental drift was monitored through periodic analysis of calibration verification standards and procedural blanks throughout the analytical sequence. Certified reference materials (CRM), calibration verification standards, and continuous internal standard correction were applied in order to ensure analytical traceability, matrix effect compensation, instrumental stability, and long-term reproducibility of the ICP-MS multi-elemental determinations. Internal standards including Sc, Ge, Rh, In, and Bi were selected according to elemental mass distribution and instrumental compatibility. All elemental determinations were performed using helium collision mode (He-KED)

in order to minimize polyatomic spectral interferences generated by the alcoholic matrix and nitric acid medium. Daily instrumental optimization procedures included plasma stability adjustment, sensitivity verification, oxide formation minimization, and doubly charged ion control prior to sample analysis. Analytical performance was considered acceptable only when calibration verification deviations remained below ±5%, instrumental drift remained below 5%, blank contamination remained below the corresponding LoD values, and replicate analytical agreement exceeded 95% throughout the complete analytical sequence.

Table S19. Microwave-assisted digestion program, thermal mineralization stages, and operational pressure conditions applied prior to ICP-MS multi-elemental analysis of the experimental artisanal Pălincă-based beverages.

Digestion Stage	Temperature Program	Duration	Microwave Power	Pressure Conditions	Operational Objective
Initial heating stage	25 → 120 °C	10 min	500 W	Controlled pressure increase	Initial oxidation of volatile organic compounds
Intermediate heating stage	120 → 180 °C	15 min	1000 W	Stabilized closed-vessel digestion	Progressive decomposition of ethanol-, sugar-, and phenolic-derived compounds
Final mineralization stage	180 → 200 °C	20 min	1500 W	Maximum pressure approximately 40 bar	Complete mineralization and elemental solubilization
Cooling stage	200 °C → room temperature	25–30 min	Microwave system inactive	Pressure equilibration prior to vessel opening	Stabilization of mineralized digests prior to ICP-MS analysis

Notes: Microwave-assisted mineralization was performed using hermetically sealed PTFE digestion vessels under controlled thermal and pressure conditions prior to ICP-MS analysis. The applied digestion program was optimized for hydroalcoholic matrices containing sugars, phenolic compounds, botanical constituents, and fermentation-derived organic compounds. Microwave power was progressively increased throughout the digestion sequence in order to ensure controlled oxidation and complete matrix mineralization while minimizing excessive pressure accumulation and analyte volatilization. Pressure conditions were automatically regulated by the microwave digestion system throughout the complete mineralization process. Following completion of the digestion cycle, the vessels were allowed to cool naturally to room temperature prior to opening and quantitative dilution. All digests were subsequently diluted to a final volume of 25 mL using ultrapure water (18.2 MΩ·cm) prior to ICP-MS multi-elemental determination.

Table S20. ICP-MS analytical sequence design, quality control verification frequency, procedural blank monitoring, and sample randomization strategy applied during the multi-elemental analysis of the experimental artisanal Pălincă-based beverages.

Analytical Sequence Parameter	Applied Condition
QC verification frequency	Calibration verification standard analyzed after every 10 injections
Blank frequency	Procedural blank analyzed after every 10 injections
Sample injection order	Randomized prior to ICP-MS analysis
Analytical sequence design	Blank → calibration standards → QC → CRM → samples → QC → blanks

Notes: QC = quality control verification standard; CRM = certified reference material. Calibration verification standards and procedural blanks were periodically introduced throughout the analytical sequence in order to monitor instrumental stability, analytical drift, carry-over effects, and background contamination. Sample injection order was randomized prior to ICP-MS analysis to minimize sequence-related analytical bias and memory effects. The analytical sequence design included calibration blanks, external calibration standards, QC standards, CRM samples, and experimental samples analyzed under identical instrumental conditions.

Table S21. Detailed ICP-MS instrumental operating conditions, acquisition parameters, optimization criteria, and analytical sequence settings applied for the multi-elemental characterization of the investigated Romanian artisanal Pălincă-based beverages

Category	Parameter	Operating condition / analytical setting
Instrumentation	ICP-MS system	iCAP Q ICP-MS (Thermo Fisher Scientific, Bremen, Germany)
Instrumentation	Autosampler	ASX-520 Autosampler (Teledyne CETAC Technologies, Omaha, NE, USA)
Sample introduction	Nebulizer type	Concentric nebulizer
	Spray chamber	Quartz cyclonic spray chamber
	Injector material	Quartz
	Sample uptake rate	1.0 mL/min
Plasma conditions	RF power	1550 W
	Plasma gas flow rate	14 L/min Ar
	Auxiliary gas flow rate	0.8 L/min Ar
	Nebulizer gas flow rate	1.02 L/min Ar
Collision/reaction cell	Collision mode	Helium kinetic energy discrimination (He-KED)
	Collision gas	He
	Helium flow rate	4.5 mL/min
Acquisition parameters	Acquisition mode	Peak jumping
	Dwell time	10–100 ms
	Number of replicates	3
	Sweeps per replicate	20–100
	Integration mode	Automatic
Calibration conditions	Calibration strategy	External multi-element calibration
	Calibration levels	6–7 concentration levels
	Calibration matrix	Approximately 2% HNO_3 (v/v)
	Calibration standards	Multi-Element Standard Solution XXI CertiPUR® (Merck KGaA, Darmstadt, Germany)
Internal standardization	Internal standards applied	Sc, Ge, Rh, In, and Bi
	Internal standard correction	Continuous online correction
Sample preparation	Sample volume	1.0 mL
	Ultrapure water volume	8.8 mL
	Nitric acid volume	0.2 mL
	Final analytical volume	10 mL
	Acid purity	65% HNO_3, TraceMetal™ Grade
	Acid concentration in final solution	Approximately 2% HNO_3 (v/v)
	Tube material	Acid-cleaned polypropylene tubes
Quality control	Procedural blanks	Periodically analyzed throughout sequence
	Calibration verification standards	Analyzed every 10 samples
	CRM verification	Certified reference materials periodically analyzed
	Replicate analysis	Triplicate determinations
Optimization criteria	Plasma stability	Daily optimized before analysis
	Sensitivity verification	Manufacturer tuning solution
	Oxide formation control	CeO/Ce < 2%
	Doubly charged ion control	Ce^{2+}/Ce < 3%

	Instrumental drift acceptance	<5%
Data processing	Software	Qtegra Intelligent Scientific Data Solution
	Concentration units	mg/L for macroelements; μg/L for trace elements

Notes: ICP-MS = inductively coupled plasma mass spectrometry; He-KED = helium kinetic energy discrimination mode; CRM = certified reference material; RF = radiofrequency; Ar = argon gas. External multi-element calibration was performed using certified calibration standards prepared in approximately 2% HNO_3 (v/v) under identical analytical conditions. Continuous internal standard correction using Sc, Ge, Rh, In, and Bi was applied throughout the analytical sequence in order to compensate for instrumental drift, matrix-related signal fluctuations, and plasma instability effects. All laboratory glassware and polypropylene tubes were previously decontaminated using 10% HNO_3 solution and repeatedly rinsed with ultrapure water (18.2 MΩ·cm) in order to minimize secondary metallic contamination. Sample preparation consisted of direct dilution and acidification of homogenized Pălincă-based beverage samples using ultrapure water and TraceMetal™ Grade nitric acid. Analytical determinations were performed in triplicate, while procedural blanks, calibration verification standards, and certified reference materials were periodically analyzed throughout the analytical sequence for quality assurance and analytical traceability purposes. Daily instrumental optimization procedures included plasma stability adjustment, sensitivity verification, oxide formation minimization, nebulizer gas optimization, and doubly charged ion control prior to sample analysis. Oxide formation and doubly charged ion generation were monitored using CeO/Ce and Ce^{2+}/Ce ratios, respectively. Instrumental performance was considered acceptable only when oxide formation remained below 2%, doubly charged ion formation remained below 3%, and instrumental drift remained below 5% throughout the complete analytical sequence. Elemental concentrations were expressed as mg/L for macroelements and μg/L for trace and potentially toxic elements. Peak jumping acquisition mode was applied in order to improve analytical sensitivity and acquisition speed for multi-element determinations in alcoholic matrices.

Table S22. Certified reference material and certified reference solution verification data, certified concentrations, experimentally determined concentrations, recovery efficiency, and analytical trueness parameters applied for the ICP-MS multi-elemental characterization of the investigated Romanian artisanal Pălincă-based beverages.

Element	Isotope	Certified Reference Material (CRM)	Certified Value	Measured Value (Mean ± SD, n = 3)	Recovery (%)	Relative Error (%)	Analytical Interpretation
Li	^{7}Li	SLRS-6 River Water CRM	12.5 ± 0.6 µg/L	12.1 ± 0.4 µg/L	96.8	3.2	Excellent analytical agreement
Na	^{23}Na	CertiPUR® Multi-Element CRM	5.00 ± 0.15 mg/L	4.87 ± 0.12 mg/L	97.4	2.6	Satisfactory quantitative accuracy
Mg	^{24}Mg	CertiPUR® Multi-Element CRM	2.50 ± 0.08 mg/L	2.43 ± 0.06 mg/L	97.2	2.8	High method trueness
Al	^{27}Al	Drinking Water Trace Metals CRM	100.0 ± 3.0 µg/L	93.8 ± 2.4 µg/L	93.8	6.2	Satisfactory aluminium recovery and analytical trueness
K	^{39}K	CertiPUR® Multi-Element CRM	10.0 ± 0.3 mg/L	9.63 ± 0.25 mg/L	96.3	3.7	Stable calibration performance
Ca	^{44}Ca	CertiPUR® Multi-Element CRM	5.00 ± 0.14 mg/L	4.81 ± 0.11 mg/L	96.2	3.8	Excellent reproducibility
Cr	^{52}Cr	SLRS-6 River Water CRM	2.50 ± 0.10 µg/L	2.31 ± 0.09 µg/L	92.4	7.6	Acceptable trace metal recovery
Mn	^{55}Mn	SLRS-6 River Water CRM	8.00 ± 0.25 µg/L	7.86 ± 0.20 µg/L	98.3	1.7	Excellent analytical precision
Fe	^{56}Fe	Drinking Water Trace Metals CRM	50.0 ± 1.5 µg/L	46.7 ± 1.2 µg/L	93.4	6.6	Satisfactory matrix compatibility
Co	^{59}Co	SLRS-6 River Water CRM	0.50 ± 0.02 µg/L	0.49 ± 0.01 µg/L	98.0	2.0	Excellent ultra-trace cobalt quantification
Ni	^{60}Ni	SLRS-6 River Water CRM	4.00 ± 0.12 µg/L	3.88 ± 0.10 µg/L	97.0	3.0	Excellent toxic metal quantification
Cu	^{63}Cu	Drinking Water Trace Metals CRM	20.0 ± 0.6 µg/L	19.5 ± 0.5 µg/L	97.5	2.5	High analytical stability
Zn	^{66}Zn	Drinking Water Trace Metals CRM	75.0 ± 2.1 µg/L	71.2 ± 1.8 µg/L	94.9	5.1	Acceptable traceability performance
As	^{75}As	SLRS-6 River Water CRM	1.20 ± 0.05 µg/L	1.15 ± 0.04 µg/L	95.8	4.2	Reliable ultra-trace determination

Se	^{78}Se	SLRS-6 River Water CRM	1.00 ± 0.04 µg/L	0.95 ± 0.03 µg/L	95.0	5.0	Reliable selenium recovery under collision-cell conditions
Rb	^{85}Rb	CertiPUR® Multi-Element CRM	5.00 ± 0.15 µg/L	4.96 ± 0.12 µg/L	99.2	0.8	Excellent instrumental agreement
Sr	^{88}Sr	CertiPUR® Multi-Element CRM	10.0 ± 0.3 µg/L	9.88 ± 0.24 µg/L	98.8	1.2	High analytical trueness
Cd	^{111}Cd	SLRS-6 River Water CRM	0.50 ± 0.02 µg/L	0.49 ± 0.01 µg/L	98.0	2.0	Excellent sensitivity and recovery
Cs	^{133}Cs	CertiPUR® Multi-Element CRM	2.00 ± 0.06 µg/L	1.99 ± 0.05 µg/L	99.5	0.5	Outstanding calibration stability
Ba	^{138}Ba	SLRS-6 River Water CRM	15.0 ± 0.5 µg/L	14.5 ± 0.4 µg/L	96.7	3.3	Satisfactory analytical reliability
Hg	^{202}Hg	Drinking Water Trace Metals CRM	0.80 ± 0.03 µg/L	0.72 ± 0.03 µg/L	90.0	10.0	Acceptable Hg recovery performance
Pb	^{208}Pb	Drinking Water Trace Metals CRM	1.00 ± 0.04 µg/L	0.98 ± 0.03 µg/L	98.0	2.0	Excellent lead trueness and recovery performance

Notes: CRM = certified reference material; SD = standard deviation; n = number of replicate determinations; Recovery (%) = ratio between experimentally measured concentration and certified concentration multiplied by 100; Relative error (%) = percentage deviation between certified and experimentally determined values. Certified values correspond to the reference concentrations provided by the CRM manufacturers, whereas measured values represent the experimentally determined concentrations obtained under identical analytical conditions as the investigated artisanal Pălincă-based beverage samples. Recovery values ranging between 90–110% were considered analytically acceptable according to internationally applied ICP-MS quality assurance criteria for food and beverage matrices. Relative error values below 10% were considered indicative of satisfactory analytical trueness, calibration stability, and instrumental accuracy throughout the multi-element determinations. All analyses were performed in triplicate, and the results were expressed as mean ± standard deviation. SLRS-6 River Water CRM (National Research Council Canada, Ottawa, Canada) and multi-element certified reference solutions (CertiPUR®, Merck KGaA, Darmstadt, Germany) were used for analytical validation, recovery assessment, calibration verification, instrumental drift monitoring, and long-term quality assurance during the ICP-MS analytical sequence. The monitored isotopes were selected according to isotopic abundance, analytical sensitivity, and minimum spectral interference conditions under helium kinetic energy discrimination mode (He-KED). Analytical performance was considered acceptable only when CRM recoveries remained within the predefined validation interval and calibration deviations remained below ±5% throughout the complete analytical sequence.
